# Proteomics Analysis of Lipid Droplets from the Oleaginous Alga *Chromochloris zofingiensis* Reveals Novel Proteins for Lipid Metabolism

**DOI:** 10.1016/j.gpb.2019.01.003

**Published:** 2019-09-05

**Authors:** Xiaofei Wang, Hehong Wei, Xuemei Mao, Jin Liu

**Affiliations:** Laboratory for Algae Biotechnology & Innovation, College of Engineering, Peking University, Beijing 100871, China

**Keywords:** Lipid droplet, Caleosin, Proteomics, Triacylglycerol, Lipase

## Abstract

*Chromochloris zofingiensis* represents an industrially relevant and unique green alga, given its capability of synthesizing **triacylglycerol** (TAG) and astaxanthin simultaneously for storage in **lipid droplets** (LDs). To further decipher lipid metabolism, the nitrogen deprivation (ND)-induced LDs from *C. zofingiensis* were isolated, purified, and subjected to proteomic analysis. Intriguingly, many *C. zofingiensis* LD proteins had no orthologs present in LD proteome of the model alga *Chlamydomonas reinhardtii*. Seven novel LD proteins (*i.e.*, two functionally unknown proteins, two **caleosins**, two **lipases**, and one l-gulonolactone oxidase) and the major LD protein (MLDP), which were all transcriptionally up-regulated by ND, were selected for further investigation. Heterologous expression in yeast demonstrated that all tested LD proteins were localized to LDs and all except the two functionally unknown proteins enabled yeast to produce more TAG. MLDP could restore the phenotype of *mldp* mutant strain and enhance TAG synthesis in wild-type strain of *C. reinhardtii*. Although MLDP and caleosins had a comparable abundance in LDs, they responded distinctly to ND at the transcriptional level. The two lipases, instead of functioning as TAG lipases, likely recycled polar lipids to support TAG synthesis. For the first time, we reported that l-gulonolactone oxidase was abundant in LDs and facilitated TAG accumulation. Moreover, we also proposed a novel working model for *C. zofingiensis* LDs. Taken together, our work unravels the unique characteristics of *C. zofingiensis* LDs and provides insights into algal LD biogenesis and TAG synthesis, which would facilitate genetic engineering of this alga for TAG improvement.

## Introduction

Algae-derived lipids, which are believed to be superior to plant oils for biofuel production, have been considered as the next-generation biodiesel feedstock and are receiving increasing interest [1], [2], [3], [4]. Nevertheless, substantial challenges remain for the cost-effective production of algae-based biodiesel [5], [6]. To improve the content and yield of lipids, efforts other than optimizing culture conditions such as genetic engineering are also needed for algae. This makes it imperative to better understand the biology and metabolism mechanism of triacylglycerol (TAG), the most energy-dense lipid class.

Algae, similar to higher plants, are thought to employ a series of enzymes to synthesize TAG for storage in lipid droplets (LDs) [7]. Generally, a LD consists of a TAG-filled hydrophobic core and an outer monolayer of polar lipids, which are decorated with structural proteins and functional enzymes [8]. Rather than serving as mere storage organelles, LDs are considered to be involved in numerous cellular processes including lipid homeostasis, energy metabolism, signaling, and trafficking [2], [7], [9]. The profiles of LD proteins, which help us understand LD biology and TAG metabolism, have been well documented by proteomic analysis for yeast [10], [11], [12], higher plant seeds [13], [14], [15], and mammalian adipose tissues [16], [17], [18]. LD proteomic analyses have also been reported for algae, including the green algae *Chlamydomonas reinhardtii* [19], [20], [21], *Dunaliella bardawil* [22], and *Lobosphaera incisa* [23], as well as the diatom *Fistulifera* sp. [24]. However, these studies mainly focused on protein profiles with a rare touch of functional characterization.

*Chromochloris zofingiensis* is a freshwater green alga previously referred to as *Chlorella zofingiensis* or *Muriella zofingiensis*. *C. zofingiensis* can grow vigorously under multiple trophic conditions and yield high biomass concentration and TAG levels [25], [26], [27], [28], [29], [30], thus representing a promising feedstock for biodiesel production. Due to its capacity of synthesizing the value-added keto-carotenoid astaxanthin, *C. zofingiensis* has been considered as a potential astaxanthin producer alternative to *Haematococcus pluvialis* [31]. Interestingly, TAG and astaxanthin accumulate in a well-coordinated manner and are both packed into LDs for storage [27], [28], [29], making *C. zofingiensis* a unique system for a better understanding of TAG metabolism, carotenogenesis, and LD biology. In particular, the genome of *C. zofingiensis* has recently been sequenced [32], which provides unprecedented opportunities for systematic omics studies of this alga.

Nitrogen deprivation (ND) is a common cue to induce TAG synthesis and LD formation in algae [7] and has been proven to be effective for *C. zofingiensis* [28], [29], [33]. In the present study, as a step to further decipher lipid metabolism, we isolated and purified LDs from ND-treated *C. zofingiensis* for proteomic analysis. A total of 295 proteins were identified, 163 out of which were detected in both replicates with at least two unique peptides. Functional classification of the *C. zofingiensis* LD proteins shows that LDs, in addition to serving as the storage depot, are involved in multiple cellular processes. The comparison of LD proteomes between *C. zofingiensis* and *C. reinhardtii* revealed many novel LD proteins. Furthermore, to verify their localization and effect on TAG synthesis, selected proteins were investigated in yeast and *Chlamydomonas*. We discuss implications of our findings on algal TAG synthesis and biotechnological applications of certain LD proteins in algae engineering.

## Results

### ND-induced TAG synthesis and LD accumulation in *C. zofingiensis*

**Figure 1:**
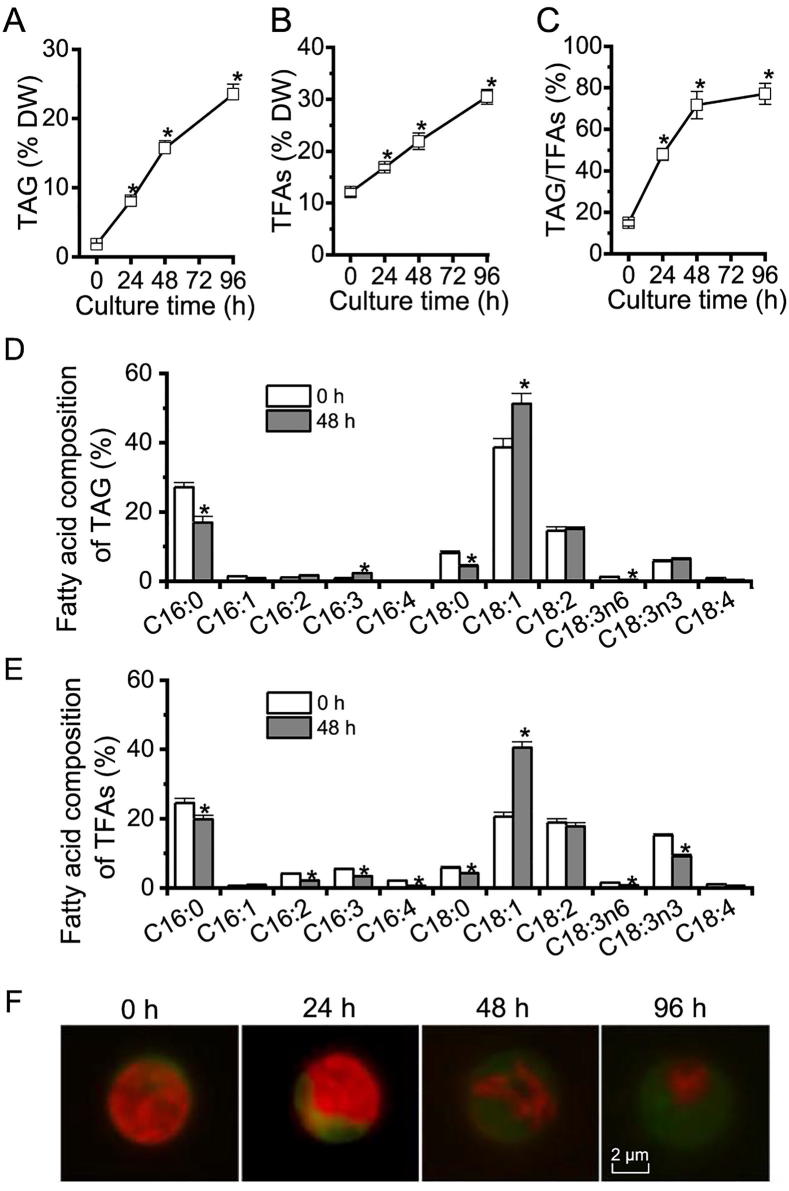
**The profiles of TAG, TFAs, and LDs in *C. zofingiensis* under ND conditions** Time course of TAG content (**A**), TFAs content (**B**), and TAG/TFAs ratio (**C**) in response to ND. Fatty acid composition of TAG (**D**) and TFAs (**E**) at 0 and 48 h of ND. **(F**) Fluorescence microscopy of cells stained with the neutral lipid dye Bodipy. LDs stained by Bodipy are shown in green, whereas red indicates chlorophyll autofluorescence. Data in A–E are expressed as mean ± SD (*n* = 3). An asterisk indicates a significant difference at the specified time point compared to 0 h (*P* < 0.05; *t-*test). TAG, triacylglycerol; TFAs, total fatty acids; LD, lipid droplet; ND, nitrogen deprivation; DW, dry weight.

Algae generally accumulate TAG under adverse conditions (*e.g.*, deprivation of nutrients particularly nitrogen) instead of vegetative growth conditions [2]. In order to evaluate TAG synthesis and LD accumulation in *C. zofingiensis*, a time course analysis was performed with respect to TAG, total fatty acids (TFAs), TAG/TFAs ratio, and LD formation in response to ND. *C. zofingiensis* synthesized only a basal level of TAG under favorable conditions (0 h, Figure 1A). Under ND conditions, TAG exhibited a sharp increase reaching 23.5% of dry weight at 96 h (Figure 1A). TFAs also showed a considerable increase, from 12.1% at 0 h to 30.6% at 96 h under ND (Figure 1B), indicative of the contribution of *de novo* fatty acid synthesis for TAG assembly. The increase of TFAs is likely attributed to TAG, as indicated by the greatly increased TAG/TFAs ratio (Figure 1C). ND also affected the fatty acid composition of both TAG and TFAs, which is characterized by an increase in C18:1 and a decrease in C16:0 (Figure 1D and E).

The LD formation under ND conditions over time is shown in Figure 1F, tracked by Bodipy, a specific fluorescence dye that binds to neutral lipids. Green fluorescence indicated LDs while red indicated the autofluorescence of chlorophylls. Under favorable growth conditions (0 h), there were almost no LDs observed (weak green signal); but under ND conditions, LDs accumulated peripherally, surrounding the shrunken chloroplast (diminished red signal). LD accumulation is positively correlated with the steady-state increase of TAG (Figure 1A), which was packed into LDs for storage.

### The purified *C. zofingiensis* LDs had minimized contamination

**Figure 2:**
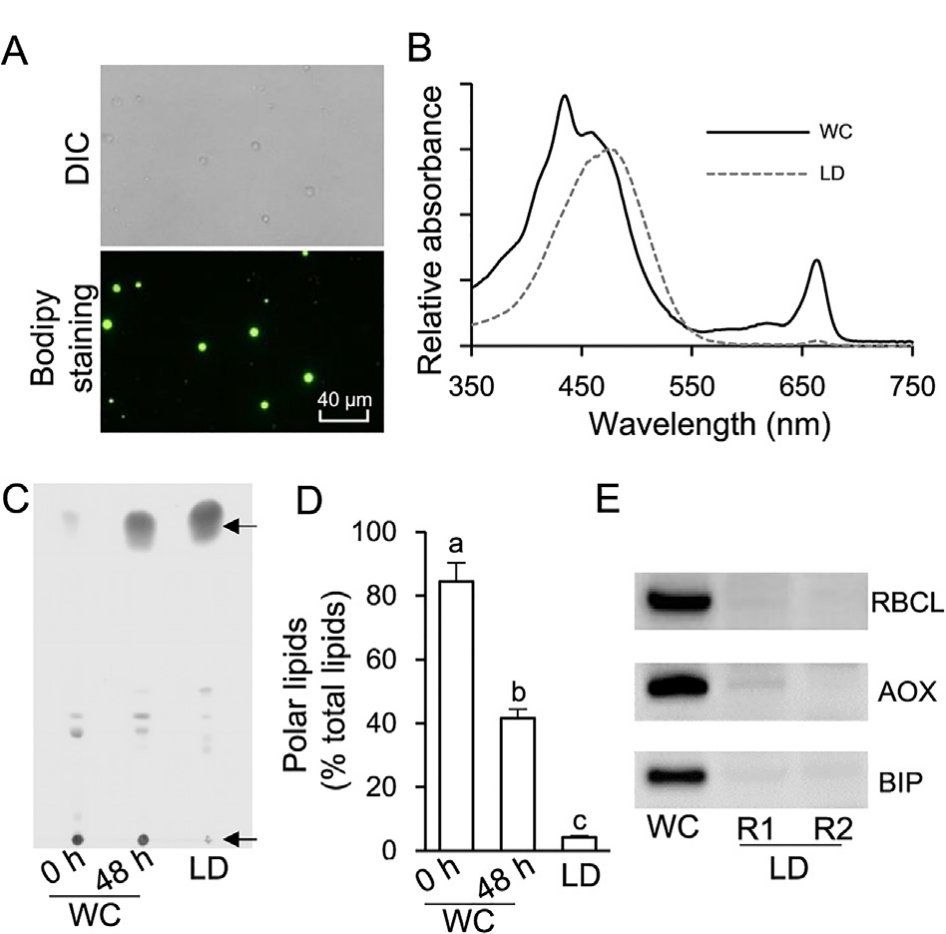
**Quality evaluation of the isolated LD fraction** **A**. Microscopic image of isolated LDs under DIC and fluorescence mode stained with Bodipy. **B.** Absorption spectra of acetone extracts obtained from ND-induced WC and purified LD fraction (48 h of ND). **C.** TLC analysis of lipid extracts from WC (0 h and 48 h of ND) and purified LD fraction (48 h of ND). Arrows on the top and bottom indicate TAG and polar lipids, respectively. **D.** The percentage of polar lipids in TFA of WC (0 h and 48 h of ND) and in purified LD fraction (48 h of ND). Data are expressed as mean ± SD (*n* = 3). Groups that are significantly different from each other are marked with different letters (*P* < 0.05; one-way ANOVA Tukey’s honestly significant difference test). **E.** Western blotting analysis for the evaluation of contamination in the LD fraction (two replicates, R1 and R2). RBCL, AOX, and BIP were used as the markers for chloroplast, mitochondria, and ER, respectively. DIC, differential interference contrast; WC, whole cell; RBCL, RuBisCO large subunit; AOX, alternative oxidase; BIP, binding immunoglobulin protein.

Cell disruption is prerequisite for preparation of the LD fraction from algae, which is not an issue for *Chlamydomonas* as a cell wall-deficient strain is available [19], [20], [21]. In contrast, *C. zofingiensis* has a thick, rigid cell wall and therefore needs a vigorous disruption process for the release of LDs. Several mechanical disruption methods have been applied to thick-walled algae for LD preparation, including oscillation with Beadbeater [24], [34], sonication [35], French Press [36], [37], and homogenization with liquid nitrogen [23], [38]. In the present study, French Press-based mechanical disruption, a relatively vigorous method, was employed to treat ND-induced *C. zofingiensis* cells. The 24-h cultures contained relatively lower amount of LDs and many more cells were needed to obtain enough LD protein, while 96-h cultures were more easily contaminated by chloroplast derivatives. Thus, the 48-h cultures were employed for LD isolation and purification. Spherical LDs were observed under both bright field and fluorescence microscopes (Figure 2A). The mechanical disruption may break intact chloroplast, resulting in contamination of LDs with chloroplast-derived pigments and proteins [36], [37]. Therefore, pigment extract from LDs was subjected to spectral scanning using the whole cell (WC) extract as a reference (Figure 2B). The results showed that compared to WC extract, LD extract had a major peak at 476 nm (keto-carotenoids) but an extremely weak peak at 663 nm (chlorophyll a), indicating the minimal contamination from chloroplast. Lipid analysis indicated that LD fraction consisted predominantly of TAG with a trace amount of polar lipids (below 5%). In contrast, compared to LD fraction, WC contained significantly more (*P* < 0.05; one-way ANOVA) polar lipids, which account for 84.4% and 41.6% at 0 h and 48 h of ND, respectively (Figure 2C and D). In addition, immunoblotting analysis was performed using three markers, namely, RuBisCO large subunit (RBCL), alternative oxidase (AOX), and binding immunoglobulin protein (BIP) targeting to chloroplast, mitochondria, and endoplasmic reticulum (ER), respectively (Figure 2E). Strong bands of RBCL, AOX, and BIP were observed for WC but not for LD fraction (two replicates, R1 and R2). These results together strongly support that the isolated LDs had minimal contamination from other organelles.

### Determination and bioinformatics analysis of proteins from purified *C. zofingiensis* LDs

**Figure 3:**
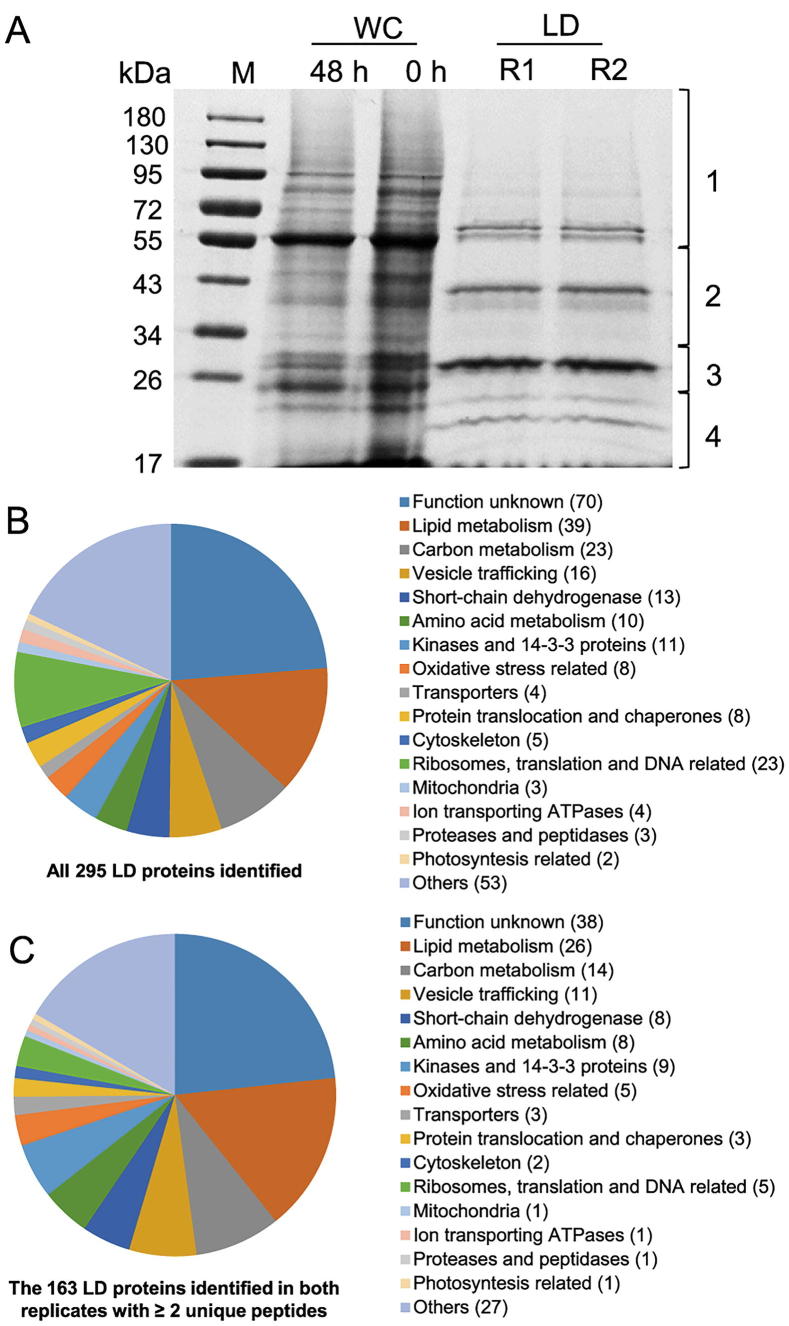
**Gel separation and functional distribution analysis of proteins from purified LDs** **A**. Proteins extracted from WC at 0 h and 48 h of ND and the LD fraction at 48 h of ND were separated using SDS-PAGE. Gel for LD fraction was sliced and numbered for proteomic analysis as indicated on the right-hand side. **B**. Functional distribution of all 295 proteins identified in LDs (see Dataset S1 for more details). **C**. Functional distribution of 163 proteins that had at least two unique peptides and were present in both replicates (see Dataset S2 for more details).

The proteins extracted from two replicates (R1 and R2) of LD fraction were separated by SDS-PAGE (Figure 3A). LD proteins, which were distinct from WC proteins, were featured by a major band of around 30 kDa and several less abundant bands. As indicated in Figure 3A, LD proteins of each replicate were excised from an SDS-PAGE gel into four fractions for LC–MS/MS analysis. In total, 295 proteins were identified, 179 (61%) of which were detected by at least two unique peptides (Figure 3B and Dataset S1). Over 90% (163 out of 179 proteins) were present in both replicates (Figure 3C and Dataset S2). Functional classification showed that *C. zofingiensis* LD proteins, excluding the function unknown group, were mainly involved in lipid metabolism, carbon metabolism, and vesicle trafficking (Figure 3C), similar to those from *Chlamydomonas* [20], [21]. By contrast, only two photosynthesis-related and mitochondria-related proteins were detected (Figure 3C), supporting the minimized contamination of our purified LDs.

Functional analysis suggested that 39 out of the 295 proteins (13%) were putatively involved in lipid metabolism, 26 of which were in both replicates with at least two unique peptides (Table 1). It is worth noting that the proteins involved in lipid metabolism accounted for 30% and 40% of the top 50 and top 10 LD proteins, respectively (Dataset S1). Cz04g29220, a homolog of the green algal major lipid droplet protein (MLDP) (Figure S1), ranked top one among the proteins involved in lipid metabolism (Table 1), as well as among all LD proteins based on the spectral count (Dataset S1). Different from oleosin, the major LD protein of higher plants [8], MLDP has no long hydrophobic segment (Figure S2) or transmembrane domain (Figure S3) and appears to be restricted to the lineage of green algae (Figure S1). Coinciding with the large increase in TAG (Figure 1), MLDP was considerably (*P* < 0.05; *t*-test) up-regulated at the transcriptional level by ND in *C. zofingiensis* (Figure S4), suggesting its structural role for stabilizing LDs as suggested in other green algae [20], [23], [34], [39]. Three caleosin related proteins (Cz16g16140, Cz09g31050, and Cz09g11210) together had a comparable spectral count to that of MLDP (Table 1). Caleosins represent a minor group of integral LD proteins (2%–4% of oleosin) in plants, and likely play roles in LD stabilization and defense against stresses [8], [40], [41]. In addition to the aforementioned structural proteins, many functional enzymes were observed, including lipases, long chain acyl-CoA synthetases, and fatty acid synthesis-related enzymes (Table 1), supporting the notion that LDs serve as not only energy storage depots but also dynamic structures for lipid and energy metabolism [7].

**Table 1:** **LD proteins putatively involved in lipid metabolism**

**LD protein**	**Protein ID**	**Unique peptides**			**Average spectral count**	**Annotation**	**Best hit in *Chlamydomonas***		**Best hit in Arabidopsis**	
		**R1**	**R2**	**Total No.**			**Protein ID**	**e-value**	**Protein ID**	**e-value**
#1	Cz04g29220	P	P	36	1651	Major lipid droplet protein (MDLP)	Cre09.g405500	1.0E–22	NA	NA
#3	Cz16g16140	P	P	42	709	Caleosin related protein	Cre06.g287000	4.0E–56	AT2G33380	3.0E–42
#6	Cz09g31050	P	P	31	471	Caleosin related protein	Cre06.g287000	3.0E–51	AT2G33380	3.0E–47
#7	Cz09g11210	P	P	24	407	Caleosin related protein	Cre01.g029600	4.0E–57	AT2G33380	1.0E–39
#13	Cz01g06170	P	P	39	137	TAG lipase (class 3)	NA	NA	AT1G45201	8.0E–12
#17	Cz05g05050	P	P	13	85	Acylglycerone-phosphate reductase	Cre12.g559350	5.0E–32	AT5G10050	4.0E–31
#23	Cz13g06240	P	P	16	55	Prostaglandin-E2 9-reductase	Cre17.g731350	2.0E–27	AT5G51030	1.0E–23
#26	Cz13g06250	P	P	18	48	Prostaglandin-E2 9-reductase	Cre17.g731350	7.0E–30	AT5G61830	7.0E–29
#36	Cz07g22230	P	P	15	27	Long chain acyl-CoA synthetase	Cre03.g182050	0.0E+00	AT4G11030	0.0E+00
#39	Cz12g10010	P	P	11	22	TAG lipase	NA	NA	AT5G42930	2.0E–11
#40	Cz04g02040	P	P	15	21	Acyl dehydratase (MaoC-like domain)	Cre06.g278196	7.0E–32	NA	NA
#41	Cz03g13150	P	P	2	20	Caleosin related protein	Cre06.g287000	1.0E–65	AT2G33380	4.0E–38
#47	Cz10g15240	P	P	3	19	Esterase/lipase	NA	NA	AT1G68620	7.0E–12
#48	Cz01g36150	P	P	15	19	Long chain acyl-CoA synthetase	Cre03.g182050	0.0E+00	AT4G23850	0.0E+00
#49	Cz02g13210	P	P	9	19	Monoglyceride lipase	Cre12.g504350	1.0E–84	AT5G16120	6.0E–36
#55	Cz05g26070	P	P	11	17	Cycloartenol synthase	Cre01.g011100	0.0E+00	AT2G07050	0.0E+00
#73	Cz07g22050	P	P	8	12	MPBQ/MSBQ methyltransferase	Cre14.g625450	2.0E–167	AT3G63410	3.0E–135
#75	Cz11g15020	P	P	7	11	Polyketide cyclase	Cre11.g467760	1.0E–18	NA	NA
#77	Cz11g20040	P	P	6	11	Enoyl-(acyl carrier protein) reductase	Cre06.g294950	4.0E–169	AT2G05990	5.0E–146
#98	Cz02g23030	-	P	6	7	Cholesterol 24-hydroxylase	Cre03.g161250	2.0E–79	AT4G12320	4.0E–31
#106	Cz13g05150	P	P	5	6	Malonyl-CoA:ACP acyltransferase	Cre14.g621650	2.0E–168	AT2G30200	2.0E–152
#109	Cz14g03070	P	P	3	6	alpha/beta hydrolase fold	Cre16.g673553	2.0E–31	AT2G36290	1.0E–18
#110	Cz01g13260	P	P	5	6	Betaine lipid synthase	Cre07.g324200	0.0E+00	NA	NA
#132	Cz01g34370	-	P	3	3	3-oxoacyl-ACP reductase	Cre03.g172000	2.0E–120	AT1G24360	8.0E–90
#137	Cz03g07200	P	P	2	3	Trans-2-enoyl-CoA reductase	Cre11.g476550	1.0E–66	AT3G45770	1.0E–24
#157	Cz02g12160	P	P	3	2	Fatty acyl-CoA reductase	Cre03.g171700	3.0E–60	AT5G22500	3.0E–23
#159	Cz14g12050	P	P	2	2	Sterol 24-C-methyltransferase	Cre12.g500500	2.0E–81	AT5G13710	3.0E–148
#161	Cz02g32040	P	P	2	2	Lysophosphatidylcholine acyltransferase	Cre05.g248150	1.0E–66	AT1G80950	3.0E–35
#171	Cz13g10110	-	P	2	2	Biotin carboxylase (ACCase)	Cre08.g359350	0.0E+00	AT5G35360	0.0E+00
#179	Cz16g00050	P	-	1	2	Beta-ketoacyl ACP reductase	Cre03.g172000	2.0E–23	AT1G24360	3.0E–24
#187	Cz04g17090	-	P	1	1	Glycerol-3-phosphate dehydrogenase (NAD)	Cre09.g387763	7.0E–142	AT2G40690	5.0E–69
#200	UNPLg00026	-	P	2	1	Patatin-like phospholipase	NA	NA	AT2G26560	5.0E–07
#212	Cz09g17170	P	-	1	1	Phospholipase A2	Cre03.g195500	1.0E–22	AT1G33270	2.0E–20
#223	Cz03g04160	-	P	1	1	Very-long-chain beta-ketoacyl ACP synthase	Cre07.g320550	6.0E–103	AT1G01120	1.0E–80
#224	Cz03g08090	-	P	1	1	Pyruvate dehydrogenase E1-alpha	Cre02.g099850	0.0E+00	AT1G01090	0.0E+00
#228	Cz03g28270	-	P	1	1	Biotin carboxyl carrier protein (ACCase)	Cre17.g715250	8.0E–41	AT5G15530	8.0E–27
#252	Cz09g30220	-	P	1	1	Acyl-carrier protein (ACP)	Cre13.g577100	2.0E–37	AT3G05020	5.0E–21
#254	Cz11g20120	-	P	1	1	Long chain acyl-CoA synthetase	Cre13.g566650	0.0E+00	AT4G23850	0.0E+00
#280	Cz04g13030	P	-	1	1	Phosphatidylethanolamine-binding protein	NA	NA	AT4G20370	3.0E–25

*Note*: The serial numbers of LD proteins in first column are from Dataset S1 and indicate the order of average spectral count (from highest to lowest) for all 295 LD proteins. The average spectral count was calculated based on two replicates (R1 and R2). Best hit proteins of *C. reinhardtii* and their e-values are provided according to v5.5, https://phytozome.jgi.doe.gov/pz/portal.html#!info?alias=Org_Creinhardtii, whereas the best hit proteins of Arabidopsis and their e-values are provided according to TAIR10, https://phytozome.jgi.doe.gov/pz/portal.html#!info?alias=Org_Athaliana. NA, not available; P, present; -, not detected

*C. zofingiensis* LDs also contained many unknown proteins, *e.g.*, Cz05g02110 (#2), Cz01g08270 (#4), and Cz10g23260 (#9), which represented the most abundant proteins within the function unknown group (Datasets S1 and S2). They may function as novel structural proteins of LDs. Besides, there were high-abundance proteins with functions other than lipid metabolism, for example, two putative retinol dehydrogenases/short-chain dehydrogenases, Cz02g13230 (#8) and Cz07g13040 (#10), and one l-gulonolactone oxidase (GULO) (Cz07g32180 (#5)). *C. zofingiensis* synthesizes carotenoid for storage in LDs [31], where retinol dehydrogenases are present possibly for carotenoid metabolism, similar to *Dunaliella* [22]. The presence of abundant GULO in LD proteome is particularly interesting, which has not been reported in other algae and appears to be unique to *C. zofingiensis*.

### Heterologous expression of selected *C. zofingiensis* LD proteins in yeast confirmed their subcellular localization

**Figure 4:**
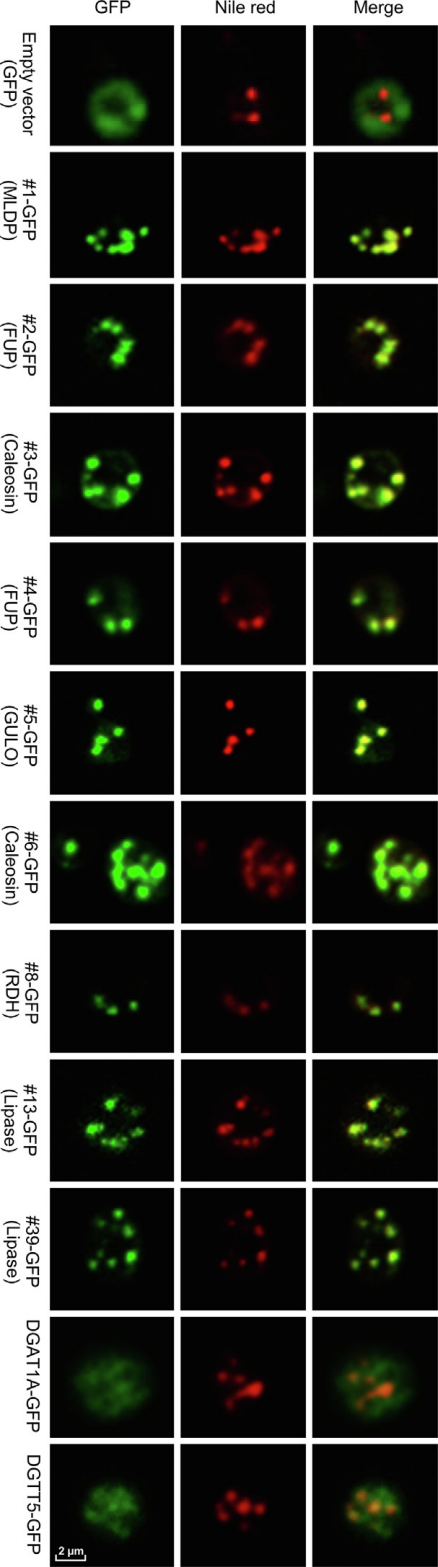
**Subcellular localization of *C. zofingiensis* LD proteins in yeast cells** The selected genes fused upstream of GFP were introduced into yeast cells and incubated with galactose for 48 h to induce gene expression. The empty vector (GFP only) was included as a control, GFP fluorescence is shown in green, and red indicates LDs stained with Nile red, a specific fluorescent dye for LDs. A representative image was shown for each transformant. More information about the LD proteins examined is available in Dataset S1. MLDP, major lipid droplet protein; FUP, functionally unknown protein; GULO, l-gulonolactone oxidase; RDH, retinol dehydrogenase; DGAT1A, type I diacylglycerol acyltransferase 1A; DGTT5, type II diacylglycerol acyltransferase 5. DGAT1A and DGTT5, which are not present in LD proteome, were used as negative controls.

To confirm the localization of LD proteins, a C-terminally tagged GFP fusion protein system was employed. As such a system is so far not available in *C. zofingiensis*, we chose the model yeast *Saccharomyces cerevisiae* for experiments, which has been frequently used to validate the LD localization of heterologous proteins from organisms like higher plants and algae [42], [43], [44]. A total of nine LD proteins were examined, including MLDP (#1), two functionally unknown proteins (#2 and #4), two caleosin proteins (#3 and #6), GULO (#5), a retinol dehydrogenase/short-chain dehydrogenase (#8), and two lipases (#13 and #39). The yeast cells transformed with GFP alone served as control with green fluorescence observed in the cytosol, which was distinct from the pattern of Nile red staining of LDs (Figure 4). By contrast, all the nine proteins examined, when expressed in yeast cells tagged with GFP, exhibited a perfect overlap of green and red signals (Figure 4), confirming their localization in LDs. We also examined the localization of a type I diacylglycerol acyltransferase 1A (DGAT1A) and a type II diacylglycerol acyltransferase 5 (DGTT5) [45], which were not detected in our LD proteome (Dataset S1), to exclude the possibility of false positive observation introduced by GFP fusion. These two diacylglycerol acetyltransferases were not localized in LDs, but rather likely at the endoplasmic reticulum (ER) (Figure 4), demonstrating that GFP fusion proteins expressed in yeast can be used to confirm the subcellular localization of LD proteins. The confirmation of all selected proteins in LDs further supported that our LD fraction was of high quality and had minimized contamination. It is worth noting that although both yeast and algae are eukaryotic organisms, algal cells contain the chloroplast organelle, which is not present in yeast cells. We cannot exclude the possibility that some LD proteins of *C. zofingiensis* may have additional localization sites such as the chloroplast envelop.

### Heterologous expression of *C. zofingiensis* LD protein-coding genes promoted TAG synthesis in yeast

It has been reported that expression of certain LD proteins facilitates TAG synthesis and LD formation in different hosts [35], [42], [46]. Nile red staining analysis suggested the expression of most examined LD proteins in yeast cells led to the formation of more LDs (Figure 4). In order to examine the effect of these proteins on TAG synthesis, the TAG content was quantified by measuring the fatty acid methyl esters using gas chromatography-mass spectrometry (GC–MS). The expression of all proteins except #2, #4, and #8 led to a significant TAG increase (*P* < 0.05), reaching ∼55% more TAG (#12) than the control (Table 2). Although there is no change in TAG content in the yeast cells expressing #2 or #4, the fatty acid composition was affected in a similar pattern to that in yeast cells expressing #1, #3, #5, or #6. The abundance of saturated fatty acids increased at the expense of that of unsaturated fatty acids (Table 2). By contrast, compared to control, the yeast cells expressing lipases (#13 and #39) exhibited little change in the fatty acid profile of TAG.

**Table 2:** **The content and fatty acid composition of TAG in yeast as affected by the expression of LD proteins**

**LD protein**	**TAG content** **(µg/OD_600_)**	**Fatty acid composition of TAG (%)**			
		**C16:0**	**C16:1**	**C18:0**	**C18:1**
Empty vector	12.95 ± 0.58	29.40 ± 0.85	33.45 ± 1.38	14.87 ± 0.57	22.29 ± 0.17
#1 (MLDP)	16.71 ± 1.03*	32.43 ± 0.95*	29.72 ± 1.16*	17.26 ± 0.76*	20.59 ± 1.22
#2 (FUP)	10.74 ± 1.04	35.92 ± 0.72*	28.57 ± 0.28*	17.28 ± 0.27*	18.22 ± 0.61*
#3 (Caleosin)	16.62 ± 0.27*	40.05 ± 1.63*	23.32 ± 0.72*	20.13 ± 0.26*	16.50 ± 1.38*
#4 (FUP)	13.99 ± 0.34	39.77 ± 0.62*	22.28 ± 2.15*	20.73 ± 0.44*	17.22 ± 2.18*
#5 (GULO)	17.98 ± 0.97*	35.25 ± 0.66*	27.97 ± 1.30*	17.11 ± 0.83*	19.67 ± 0.30*
#6 (Caleosin)	19.05 ± 0.99*	37.54 ± 0.96*	23.83 ± 4.00*	20.45 ± 2.31*	18.18 ± 1.12*
#8 (RDH)	11.17 ± 0.83	31.48 ± 0.83	37.26 ± 1.11*	11.31 ± 0.96*	19.95 ± 0.54*
#13 (Lipase)	20.08 ± 1.01*	30.43 ± 0.47	30.59 ± 0.98	14.06 ± 1.31	24.92 ± 0.50
#39 (Lipase)	17.57 ± 0.61*	28.66 ± 1.29	33.92 ± 1.20	12.78 ± 0.57*	24.64 ± 0.94

*Note*: Data are expressed as mean ± SD (*n* = 3); Asterisk indicates the significant difference compared to the empty vector control (*P* < 0.05; *t*-test). See Dataset S1 for more information about the LD proteins shown in the first column. TAG content was expressed as µg/OD_600_ in yeast cells. MLDP, major lipid droplet protein; FUP, function unknown protein; GULO, l-gulonolactone oxidase; OD, optical density; RDH, retinol dehydrogenase.

### *C. zofingiensis MLDP* rescues the phenotype of *C. reinhardtii mldp* mutant and enhanced TAG synthesis

**Figure 5:**
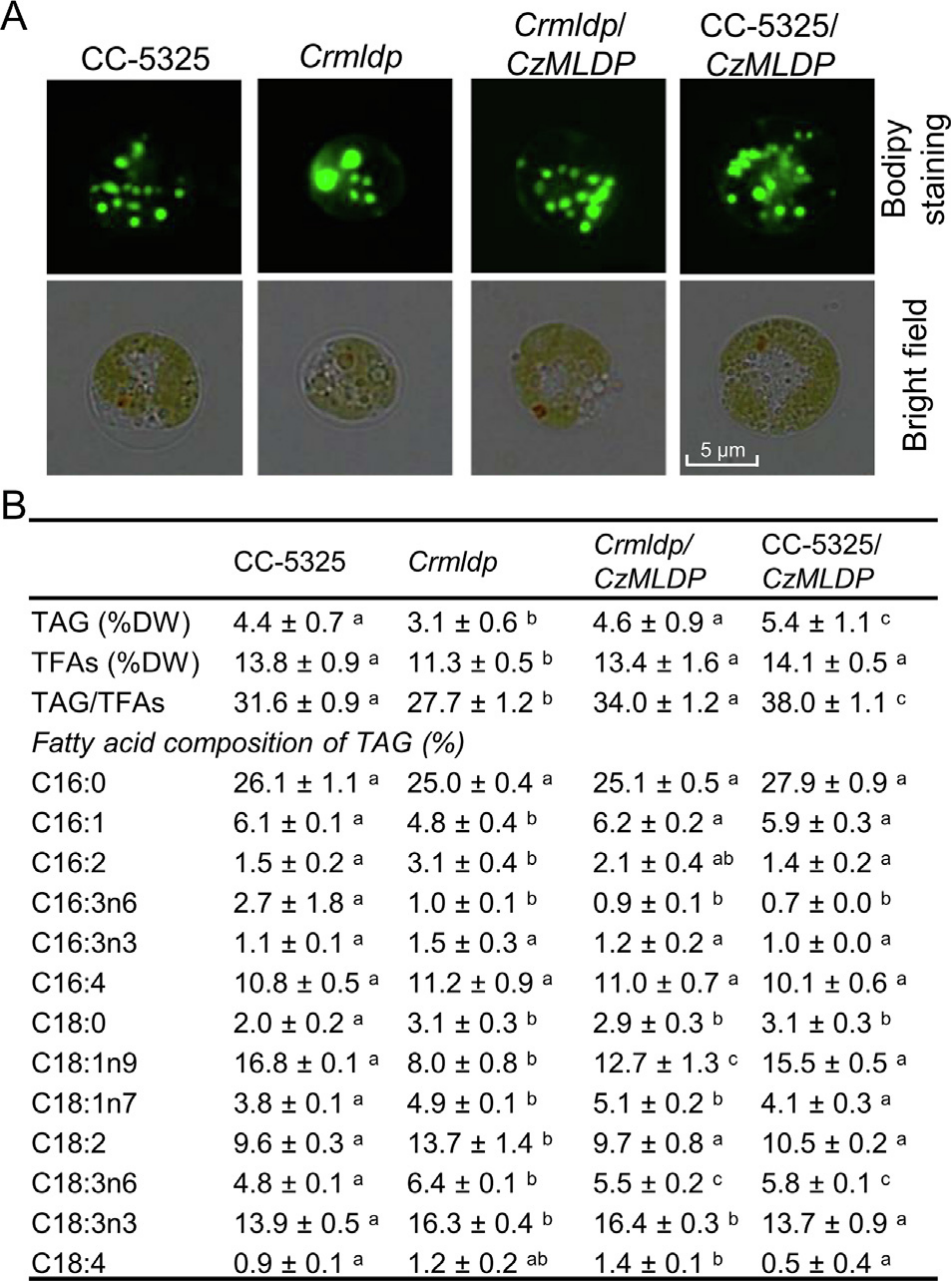
**Heterologous expression of *C. zofingiensis MLDP* in *C. reinhardtii*** **A.** Microscopic visualization of LDs (indicated by green signal) in various *Chlamydomonas* strains of CC-5325, *Crmldp*, *Crmldp*/*CzMLDP*, and CC-5325/*CzMLDP*. **B.** Contents of TAG and TFAs, TAG/TFAs ratio, and fatty acid profile of TAG. Algal cells were grown under ND conditions for 48 h. Data are expressed as mean ± SD (*n* = 3). Groups that are significantly different from each other are marked with different letters, whereas no significant difference was found between groups labelled with the same letters (*P* < 0.05; one-way ANOVA Tukey’s honestly significant difference test). *Cz*, *C. zofingiensis*; *Cr*, *Chlamydomonas reinhardtii*; *Crmldp*/*CzMLDP*, the *Crmldp* mutant strain expressing *CzMLDP* gene; CC-5325/*CzMLDP*, the wild-type strain CC-5325 expressing *CzMLDP* gene.

It has been demonstrated in *C. reinhardtii* that MLDP is involved in maintaining LD size and preventing LD coalescence [20], [47]. However, the role of MLDP orthologs from non-model algae, particularly industrially relevant strains, remains unknown and awaits experimental validation. To address the function of *C. zofingiensis* MLDP (CzMLDP), we optimized the codon usage of *CzMLDP* based on *C. reinhardtii* codon bias and introduced it into a *C. reinhardtii mldp* mutant (*Crmldp*, with insertional disruption in the *MLDP* gene). The *Crmldp* mutant contained fewer, larger-sized LDs (Figure 5A), and significantly (*P* < 0.05) lower levels of TAG and TAG/TFAs ratios than its parental strain CC-5325 (Figure 5B). Introducing *CzMLDP* gene into the *Crmldp* mutant led to the formation of more, smaller-sized LDs, and an significant (*P* < 0.05) increase in TAG levels and TAG/TFAs ratios (Figure 5A and B). In addition, the *MLDP* mutation in *C. reinhardtii* impacted the fatty acid profile of TAG, that is, increasing the percentage of monounsaturated fatty acids (C16:1 and C18:1n9) and decreasing the percentage of polyunsaturated fatty acids (C16:2, C18:2, and C18:3). This was partially restored by the heterologous expression of *CzMLDP* (Figure 5B). These results suggest CzMLDP functions in a manner similar to *C. reinhardtii* MLDP in regulating TAG synthesis and LD biogenesis. Interestingly, the expression of *CzMLDP* in CC5325 also enhanced TAG levels, though it had little effect on the fatty acid composition of TAG and LD size (Figure 5).

## Discussion

### MLDP is the major structural protein of *C. zofingiensis* LDs with function analogous to CrMLDP

Structural proteins are required for the stabilization of LDs, such as perilipin in mammals [18] and oleosin in higher plants [8]. Algal LDs contain no proteins homologous to either perilipin or oleosin. Instead, there are unique major structural proteins present in algal LDs, including MLDP from green algae such as *C. reinhardtii* [20], [21], *H. pluvialis* [32], and *Dunaliella salina* [39], the LD surface protein from the heterokont alga *Nannochloropsis oceanica* [36], and stramenopile-type LD protein from the diatom *Phaeodactylum tricornutum* [37]. These algal structural proteins are phylogenetically distant (Figure S1). Unlike oleosin from higher plants that has a long hydrophobic segment stretching into the TAG matrix of LDs (tight association with LDs), MLDP from *C. reinhardtii* resides embracing the LDs, in a cup-shape manner rather than fully submerged [47], [48], pointing to its relatively loose association with LDs. In *C. zofingiensis*, MLDP represents the major structural protein of LDs as well (Dataset S1), and is likely localized to the surface of LDs, considering that the area of GFP signal is somewhat larger than that of LDs (Figure 4). The lack of a hydrophobic segment for inserting into the hydrophobic core of LDs indicates that MLDP may be directly attached to the polar lipid monolayer of LDs, owing to its intrinsic hydropathic and topological properties. If so, one may raise the question of what motif(s) is required for directing MLDP to LDs? MLDP protein sequences of different origin possess many conserved amino acid residues (Figure S5), which probably play a role in the association of MLDP with LDs and is worthy of future investigation. It is also possible that the attachment of MLDP to LDs is mediated by certain proteins such as microtubules as suggested by Tsai et al [47], yet more experimental evidence is needed. Nevertheless, the LD localization provides biotechnological implications in using MLDP for the production of recombinant proteins enriched in LDs, as is the case for oleosin [49].

It has been shown in *C. reinhardtii* that the knockdown of *CrMLDP* by RNA interference (RNAi) resulted in fewer LDs with increased size, but no change in TAG content [20], [47]. In the present study, the *MLDP* knockout mutant *Crmldp* also showed fewer yet larger LDs compared to its parental strain (Figure 5A). Nevertheless, the *Crmldp* strain accumulated significantly (*P* < 0.05) lower levels of TAG (Figure 5B). This difference is likely due to the different *MLDP* suppression efficiencies between these strains: the RNAi-mediated *MLDP* knockdown achieved only ∼60% suppression efficiency at the mRNA level [20], while the insertional knockout of *MLDP* led to a suppression efficiency of 89% (Figure S6). Heterologous expression of the codon-optimized *CzMLDP* rescued the phenotype of *Crmldp* including the size and number of LDs, contents of TAG and TFA, TAG/TFA ratio, and the fatty acid composition of TAG (Figure 5), strongly supporting that CzMLDP possesses functions fully analogous to CrMLDP. Furthermore, overexpression of *CzMLDP* promoted TAG content in the wild-type strain of *C. reinhardtii* (Figure 5) and in yeast (Table 2), suggesting that in addition to maintaining LD size, MLDP facilitates TAG accumulation. Our results are consistent with previous studies in which expression of LD proteins such as *Arabidopsis* oleosin [42] and seipin [50], murine perilipin [42], [51], [52], *H. pluvialis* MLDP [46], and *P. tricornutum* LD protein 1 [35] leads to increased levels of TAG in various hosts. TAG increase caused by the expression of perilipin is thought to be attributed to the attenuated TAG hydrolysis by interfering with the access of lipases to TAG [51]. However, this is unlikely the case for MLDP, as its suppression in *C. reinhardtii* resulted in a delay of TAG breakdown [47]. *MLDP* overexpression probably facilitates LD formation for more efficient sequestration of neutral lipids, thus attenuating the end-product inhibition in TAG synthesizing enzymes and promoting TAG synthesis, as proposed by Jacquier et al [42]. In fact, MLDP recruits proteins of different functions to LDs including those lipid metabolism-related enzymes [47], further supporting such novel roles as in lipid homeostasis other than only serving as a structural protein to stabilize LDs. Thus, *MLDP* also represents a potential gene target for manipulating TAG production.

### Proteomic analysis reveals novel proteins present in *C. zofingiensis* LDs

In addition to the major proteins mentioned above, LDs consist of many proteins of different functions. Protein profile analysis of algal LDs started with the model alga *C. reinhardtii* by several independent groups [19], [20], [21]. Moellering and Benning [20] identified 259 LD proteins, while Nguyen et al [21] identified 248 LD proteins. Our proteomic study of *C. zofingiensis* LDs revealed a total of 163 proteins detected in both replicates with at least two unique peptides (Figure 3 and Dataset S2), considerably fewer as compared to *C. reinhardtii* LD proteomes. This may be partially due to the minimized contamination of our purified LD fraction. Intriguingly, the comparison of LD proteomes between *C. zofingiensis* and *C. reinhardtii* revealed that 58% proteins (94/163) in *C. zofingiensis* LD proteome had no homolog found in the *C. reinhardtii* LD proteome (Dataset S2), although most of them showed hits when searching the whole protein database of *C. reinhardtii* (Dataset S1). These include many functionally unknown proteins, four caleosins, five putative lipases, and one GULO. We chose nine of them for functional investigation in yeast (Figure 4 and Table 2). Among them, #2 and #4, two functionally unknown proteins, were both localized to LDs (Figure 4), but had no effect on TAG content (Table 2). Considering that these two proteins are highly abundant in the LD proteome and lack any features of known catalytic activity, they may function merely as structural proteins.

### LD protein #5 is an LD-localized GULO with engineering potential for TAG improvement

It is believed that ascorbic acid can be derived from l-galactono-1,4-lactone catalyzed by l-galactono-1,4-lactone dehydrogenase (GLDH) and/or l-gulono-1,4-lactone catalyzed by GULO [53]. Both GULO (#5) and GLDH (Cz16g21050) were present in *C. zofingiensis* and encoded each by a single-copy gene. Intriguingly, unlike GLDH that is clustered with other green algae, *C. zofingiensis* GULO is relatively closer to the orthologs of higher plants (Figure S7). The presence of GULO in LDs, which has not been reported before, represents a unique characteristic of *C. zofingiensis* LD proteome. Similar to MLDP, GULO has no long hydrophobic segment (Figure S2) or transmembrane domain (Figure S3). Although MLDP is thought to recruit proteins to LDs in *Chlamydomonas* [47], the localization of GULO to LDs is unlikely mediated by MLDP in *C. zofingiensis*, as GULO is also targeted to LDs in yeast (Figure 4), where MLDP is absent. The overexpression of *GULO* in yeast was accompanied by the enhanced TAG accumulation (Table 2), probably attributed to the increased production of ascorbic acid, which has strong antioxidant capacity to cope with lipid peroxidation. Similarly, in *C. zofingiensis* the significant up-regulation (*P* < 0.05; *t*-test) of *GULO* transcripts (Figure S4) correlated with the great increase in TAG level (Figure 1).

### LD proteins #3 and #6 are caleosins, the major integral proteins of LDs

Caleosin is widely present in higher plants and represents a minor group of integral LD proteins (2%–4% of oleosin) [8], [54]. By contrast, although many algae possess caleosin-coding genes (Figure S1), the presence of caleosin in algal LDs has only been reported by Lin et al [38], which claimed caleosin rather than MLDP was the major protein of LDs of a *Chlorella* species. Four out of five putative caleosins in *C. zofingiensis* (Figure S1), namely Cz16g16140 (#3), Cz09g31050 (#6), Cz09g11210 (#7), and Cz03g13150 (#41), were found in the LD proteome (Dataset S2). The former three, representing the 2nd, 3rd, and 4th most abundant proteins involved in lipid metabolism, together have a total spectral count comparable to MLDP (Table 1). This suggests that *C. zofingiensis* caleosins, similar to MLDP, serve as the major group of LD structural proteins. To the best of our knowledge, we report, for the first time, that MLDP and caleosins are present in comparable abundance in the LD proteome, pointing to an additional unique characteristic of *C. zofingiensis* LDs.

Subcellular localization studies using yeast demonstrate that #3 and #6 are LD-targeted (Figure 4). The LD association of caleosin is thought to be caused by its central hydrophobic segment, which forms a hairpin and stretches into the monolayer of polar lipids for anchoring [55], and thus should be bound more tightly than MLDP. Recently, it had been demonstrated in yeast that the N-terminus of *Arabidopsis* caleosin CLO1 plays a more important role than the central hydrophobic segment in LD docking [56]. This claim, nevertheless, may need experimental validation in plants, as done for oleosin [57]. Aside from the hydrophobic segment, caleosin contains several phosphorylation sites and a conserved calcium-binding motif [40], [54], [55], indicative of additional biological functions rather than serving merely as a structure protein for the stabilization of LDs.

In our study, heterologous expression *C. zofingeinsis* caleosin-coding genes leads to higher intracellular TAG level (Table 1), implying a role of caleosin in TAG accumulation. Consistent with our results, the expression of *Arabidopsis* caleosin also promotes TAG accumulation in yeast [58]. The enhanced TAG accumulation may be ascribed to attenuated TAG degradation and/or enhanced TAG synthesis, which remains to be addressed. It is worth noting that in *C. zofingiensis* under ND conditions, the transcriptional expression of *MLDP* was up-regulated at early stages (6 h) while the transcriptional expression of caleosin genes was up-regulated only at late stages (24 h) (Figure S4). The differential temporal expression pattern, in combination with the fact that small LDs are turned into big ones at late stages of ND in *C. zofingiensis* [31], raises the possibility that MLDP and caleosin function differently *in vivo* during LD biogenesis. Probably, MLDP facilitates the formation of nascent LDs and maintains their size, while caleosin mediates the fusion of nascent LDs to produce large ones [40]. By contrast, *C. reinhardtii* LDs do not appear to coalesce to form larger droplets as in *C. zofingiensis* [19], [20], which may be explained by the absence of caleosins in the LDs [20], [21], [47]. It is interesting to note that *C. reinhardtii* genome possesses caleosin-coding genes, which are closely related to those from *C. zofingiensis* (Figure S1). However, these genes are lowly expressed even under ND conditions, as indicated by the RNA-seq data from Goodenough et al [59]. This may partially explain the non-detectable level of caleosins in *C. reinhardtii* LDs. Nevertheless, we cannot exclude the possibility that in *C. reinhardtii* caleosins may reside at other organelles such as ER, which is the case in higher plants [8].

### LD proteins #13 and #39 are likely polar lipid lipases and contribute to TAG synthesis

Lipases are involved in the metabolism of LDs. Proteomic studies have demonstrated the presence of lipases in LDs of yeast, which are mainly TAG lipases with confirmed function for TAG remobilization [10], [11]. Lipases are also present in the LDs of higher plants [15], [60] and algae [20], [21], [22], [23], but their function remains largely unknown with the exception of the TAG lipase SUGAR-DEPENDENT1 (SDP1) from *Arabidopsis* [60]. Two class 3 lipases (#13 and #39) were present in the LD proteome of *C. zofingiensis*, which were tentatively annotated as TAG lipase (Table 1). These two proteins, phylogenetically distant from SDP1 and the yeast TAG lipases, TGL3 and TGL4 (Figure S8), possess transmembrane domain(s) (Figure S3), which may anchor the proteins to the surface of LDs (Figure 4). Intriguingly, the expression of these two lipases each leads to enhanced TAG accumulation in yeast (Table 1), questioning their function for TAG lipolysis. Furthermore, the expression of these two lipase genes is significantly (*P* < 0.05; *t*-test) up-regulated by ND (Figure S4), in correlation with the ND-induced drastic increase of TAG in *C. zofingeinsis* (Figure 1). These finding suggested that they may function as lipases to recycle polar lipids for TAG synthesis rather than to hydrolyze TAG. We thus hypothesize that these two lipases act on polar lipids (LDs and/or membrane contact sites between LDs and ER and between LDs and chloroplast) for the release of free fatty acids, which are readily used by LD-localized long chain acyl-CoA synthetases (#36 and #48, Table 1) to produce acyl-CoAs and then by ER-localized diacylglycerol acyltransferases (Figure 4) to form TAG in *C. zofingiensis*. It has been demonstrated in *C. reinhardtii* that Plastid Galactoglycerolipid Degradation1 (PGD1), a galactolipase, is involved in monogalactosyl diacylglycerol (MGDG) turnover and contributes acyl chains to ND-induced TAG synthesis [61]. In *C. zofingiensis*, membrane lipids other than MGDG also decrease in response to ND [28], [33], indicative of the involvement of additional lipases in membrane lipid turnover. The proteins #13 and #39, which have no homologs present in *C. reinhardtii* (Dataset S1), may represent such membrane lipases. Likely, these two lipases, in collaboration with PGD1, recycle various membrane lipids to support TAG assembly, allowing *C. zofingiesnis* to produce more TAG than *C. reinhardtii*. Therefore, it is of particular interest to figure out substrates of these two lipases, which are worthy of future investigation and may be addressed by *in vitro* assays.

As an initial step, the current study focused examines the proteome analysis of LDs from *C. zofingiensis* cultured for 48 h under ND condition. The single-time-point analysis, however, reflects only a snapshot of the protein profile of LDs. We have no idea about the temporal dynamics of the proteins, which relies on the time-resolved proteomic analyses and remains to be explored in the future. In addition to ND, many other abiotic stresses, *e.g.*, phosphorus deprivation, sulfur deprivation, high light, and high salinity, can also induce TAG synthesis for LD formation in algae [28], [62], [63], [64]. How these stress conditions affect the protein profiles of LDs and whether there is a difference in LD proteome between these stress conditions are interesting questions and worthy of further investigation. Moreover, we characterized only nine LD proteins in yeast and/or *C. reinhardtii* in the present study. A better understanding of LD biology may lie in the characterization of these proteins *in vivo*, as well as characterization of additional LD proteins listed in Dataset S2.

## Conclusion

**Figure 6:**
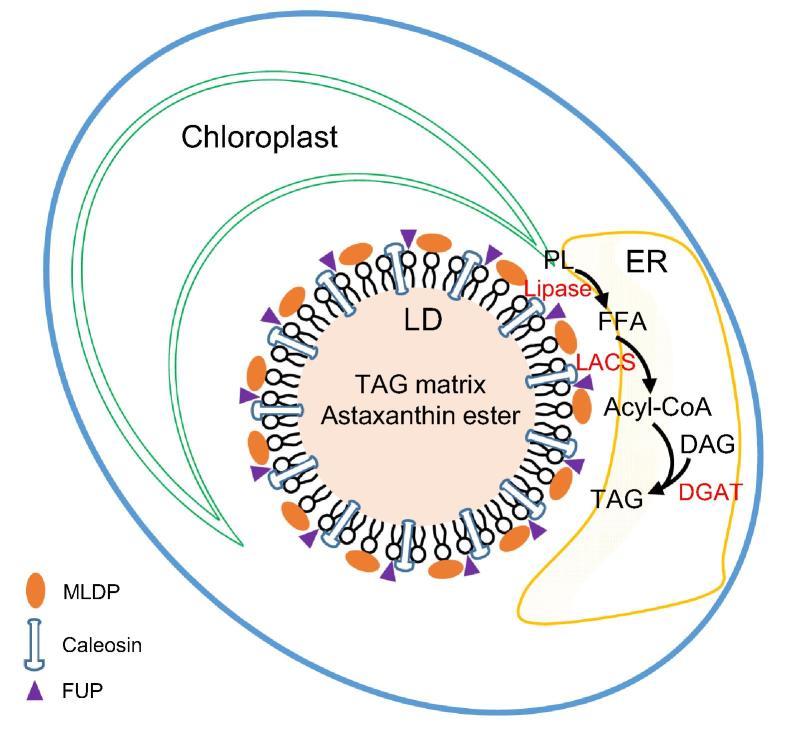
**A proposed model for *C. zofingiensis* LDs** LDs are in close contact with ER and chloroplast, enveloped and decorated with many structural proteins and functional enzymes. Structural proteins, *e.g.*, MLDP, caleosins, and certain functionally unknown proteins, are shown to be associated with the PL monolayer of LDs, maintaining the stability of LDs. Enzymes involved in lipid metabolism such as PL lipase and LACS, working in concert with other lipid metabolism-related enzymes localized in ER (*e.g.*, DGAT), contribute to lipid homeostasis. DAG; diacylglycerol; DGAT, diacylglycerol acyltransferase; MLDP, major lipid droplet protein; ER, endoplasmic reticulum; FFA, free fatty acid; FUP, functionally unknown protein; LACS, long chain acyl-CoA synthetase; LD, lipid droplet; PL, polar lipid; TAG, triacylglycerol.

To better understand lipid metabolism and LD biology, we isolated and purified LDs from ND-induced *C. zofingiensis* cells with minimized contaminants. Proteomic analysis of the purified LDs identified 295 proteins in total, of which 163 were detected in both replicates with at least two unique peptides. Functional analysis of the proteins supports the notion that LDs, in addition to serving as the energy depot, participate in multiple cellular biological processes. The comparison of LD proteomes between *C. zofingiensis* and *C. reinhardtii* reveals the presence of many novel proteins such as caleosins, functionally unknown proteins, and lipases, indicative of the unique characteristic of *C. zofingiensis* LDs. Together with the functional characterization of selected proteins in yeast and *C. reinhardtii*, we proposed a model for *C. zofingiensis* LDs as shown in Figure 6. LDs, in close contact with the ER and chloroplast, are decorated with many structural proteins and functional enzymes to contribute to homeostasis. On one hand, structural proteins such as MLDP, caleosins, and certain unknown proteins, are present in high abundance and maintain the stability of LDs; on the other hand, enzymes involved in lipid metabolism including polar lipid lipases and long chain acyl-CoA synthetases, work in concert with other lipid metabolism-related enzymes localized in ER (*e.g.*, diacylglycerol acyltransferases). Our results unravel several novel LD proteins important for lipid metabolism and provides insights into algal LD biogenesis and TAG synthesis, which will facilitate future genetic engineering of industrially relevant algae for improvements in TAG and other storage compounds.

## Materials and methods

### Algal stains and culture conditions

*Chromochloris zofingiensis* (ATCC 30412) was purchased from the American Type Culture Collection (ATCC, Rockville, MD). When starting experiments, the algal cells were inoculated from the agar plate into a 100-ml Erlenmeyer flask containing 10 ml of BG-11 medium and cultured for four days (temperature, 25 °C; orbital shaking, 150 rpm; light intensity, 30 μE/m^2^/s, continuous illumination), before transferred to 100 ml of medium in a 500-ml flask for another six days under the same conditions. Then, the cells were inoculated at 10% (v/v) into 250-ml columns and allowed to grow for four days (exponential growth phase) to prepare seed cultures (temperature, 25 °C; light intensity, 80 μE/m^2^/s, continuous illumination; aeration, 1.5% CO_2_ enriched air). For ND experiments, the algal cells in exponential phase (designated as 0 h of ND) were harvested by centrifugation, washed three times with N-free BG-11 medium, and then resuspended in the medium for growth in columns. Cells were sampled at 0, 24, 48, and 96 h of ND for lipid analysis, and at 48 h of ND for LD isolation.

*Chlamydomonas reinhardtii* strains CC-5325 and LMJ.RY0402.113424 were purchased from the *Chlamydomonas* Resource Center (St. Paul, MN), The former is a wild-type strain, whereas the latter is a mutant defective in the *MLDP* gene (designated as *Crmldp*) generated by Li et al [65]. Both strains were grown in TAP medium [66] (temperature, 23 °C; orbital shaking, 150 rpm; light intensity, 30 μE/m^2^/s, continuous illumination). For ND treatments, cells in the stationary growth phase (about 1 × 10^7^ cells/ml) were harvested by centrifugation, washed with N-free TAP medium (TAP-N), resuspended in TAP-N medium, and allowed to grow for four days.

### Isolation and purification of LDs from *C. zofingiensis*

Isolation and purification of LDs from *C. zofingeinsis* were performed as described by Yoneda et al [37] with the following modifications. (1) Cells were lysed using a French press (Spectronics Instruments, Rochester, NY) at an internal pressure of 15,000 psi. (2) The washing process with weak detergent buffer was repeated three times. (3) Following the detergent treatment, the LDs were washed three times with detergent-free Tris buffer.

### Contamination evaluation and LC–MS/MS analysis of purified LDs

The purified LD fraction was incubated in cold acetone at −20 °C overnight and centrifuged to pellet protein. The acetone extracts from LDs and whole cells were subjected to a UV–visible spectrophotometry (UV-1800, UV spectrophotometer, Shimadzu, Japan) for evaluating the contamination level from chloroplasts, using a full absorbance of 350–750-nm wavelength. The protein pellets were incubated in cold ethyl acetate at −20 °C for 2 h in order to remove any residual lipids and then dissolved in the lysis buffer described by Yoneda et al [37]. To further check the possible contamination of LDs by other organelles, immunoblotting analysis was performed as previously described [67] using three markers, RBCL, AOX, and BIP, which target to chloroplast, mitochondria, and endoplasmic reticulum (ER), respectively. The antibodies for these three proteins were purchased from Agrisera AB (Catalog Nos. AS03 037, AS06 152, and AS09 481, Vännäs, Sweden).

For proteomic analysis, LD proteins (two replicates, R1 and R2) were separated on 10% SDS-PAGE gels. Each gel lane was sliced into four pieces for in-gel protein digestion, followed by the extraction of peptides. The extracted peptides were then analyzed on a nanoflow liquid chromatography (EASY-nLC1000, Thermo Fisher Scientific, Waltham, MA) coupled with a hybrid linear ion trap-Orbitrap mass spectrometer (LTQ Orbitrap Velos, Thermo Fisher Scientific), following the procedures described by Qi et al [68]. LC–MS/MS raw files were searched using the software MASCOT 2.3.02 (Matrix Science, London, UK) against *C. zofingiensis* protein database downloaded from Phytozome (https://phytozome.jgi.doe.gov/pz/portal.html#!info?alias=Org_Czofingiensis_er). The protein abundance was assessed using a spectral counting method.

### Heterologous expression of *C. zofingiensis* LD proteins in yeast

Total RNA from *C. zofingeinsis* cells was extracted using the plant RNA extraction kit (Catalog No. 740949.50, TaKaRa, Tokyo, Japan) and reversely transcribed to cDNA with the Prime Script™ RT Master Mix (TaKaRa). The LD protein-encoding genes from *C. zofingiensis* were amplified using cDNA as template and cloned with C-terminal green fluorescence protein (GFP) into the vector pYES2-CT (Invitrogen, Carlsbad, CA). PCR primers used for cloning were listed in Table S1. The recombinant plasmids were confirmed by sequencing and then introduced into the *Saccharomyces cerevisiae* strain INVSc1 using the S.c. EasyComp Transformation Kit (Catalog No. K505001, Invitrogen). Transformants were selected on SD-uracil medium (Catalog No. 630416, TaKaRa). Single colonies carrying the plasmids were grown in SC-uracil medium containing 2% raffinose on an orbital shaker (250 rpm) at 30 °C for 24 h. To induce the expression of heterologous genes, yeast cells were cultured in SC-uracil medium containing 2% galactose, suspended to an initial OD_600_ of 0.4, and allowed to grow for 48 h.

### Heterologous expression of the *C. zofingiensis MLDP* gene in *C. reinhardtii*

The coding sequence of *C. zofingiensis MLDP* gene was codon-optimized for expression in *C. reinhardtii* and chemically synthesized by Sangon Biotech (Shanghai, China). The codon-optimized sequence was cloned into *Eco*RI/*Xho*I sites of the *Chlamydomonas* expression vector pOpt_Clover_Hyg [69]. The resulting plasmid, after linearization by *Xba*I, was introduced into CC-5325 and *Crmldp*, respectively, using the glass beads method [70]. The CC-5325 transformants were selected on TAP plates containing 10 μg/ml hygromycin, while *Crmldp* transformants were selected with 5 μg/ml hygromycin and 5 μg/ml paromomycin. The integration of the transgenes into genome was confirmed by PCR.

### Lipid extraction and analysis

Lipids were extracted with a chloroform/methanol method as previously described [67]. Prior to lipid extraction, *C. zofingiensis* cells were disrupted by homogenization in the presence of liquid nitrogen, while yeast cells were disrupted by glass beads in a mini bead-beater (BioSpec Products, Bartlesville, OK). Neutral lipids were separated on Silica gel 60 TLC plates (Merck, Darmstadt, Germany) and detected by charring following the procedures described by Liu et al [67]. For quantification, individual lipids on the TLC plate, after visualization with iodine vapor, were recovered and transesterified to fatty acid methyl esters (FAMEs) using 1% sulfuric acid in methanol [67]. FAMEs were analyzed by GC–MS using the Agilent 7890 capillary gas chromatograph equipped with a 5975 C mass spectrometry detector and a HP-88 capillary column (60 m × 0.25 mm) (Agilent Technologies, Santa Clara, CA), following the procedures from Liu et al [28] except using a split ratio of 19:1. In addition, C17:0 was used as the internal standard for quantification.

### Fluorescent and confocal microscopy analyses

*C. zofingiensis* and *C. reinhardtii* cells were stained with the fluorescence BODIPY™ 505/515 (Catalog No. D3921, Invitrogen) at a working concentration of 1 µg/ml, and observed under an Olympus BX51 Fluorescence Microscope (Olympus, Tokyo, Japan). Yeast cells transformed with GFP-containing plasmids were stained with Nile red (Sigma-Aldrich) at a working concentration of 0.3 µg/ml, and observed under the confocal laser-scanning microscope Nikon A1R (Nikon, Japan). Nile red fluorescence was detected with excitation at 488 nm and emission between 560 and 615 nm, and GFP signal was detected with excitation at 488 nm and emission between 500 and 530 nm.

### Bioinformatics analyses

Protein sequence alignment was performed with ClustalX2.1 [71]. Phylogenetic tree was constructed by MEGA6.0 using the neighbor-joining method [72]. Hydropathy plots were generated with ProtScale (https://web.expasy.org/protscale) employing the Kyte-Doolittle algorithm. The grand average of hydropathy (GRAVY) values were calculated using the GRAVY calculator (http://www.gravy-calculator.de). The prediction of transmembrane domains was conducted using TMHMM Server v. 2.0 (http://www.cbs.dtu.dk/services/TMHMM/).

### Statistical analysis

Data were expressed as mean ± SD (*n* = 3). SPSS was used for statistical analysis, with *t*-test for two group means and one-way ANOVA Tukey’s honestly significant difference test for more than two group means. Differences were considered statistically significant with *P* < 0.05.

## Authors’ contributions

JL and XW designed the experiments. XW, HW, XM, and JL conducted the experiments. JL and XW analyzed the data and wrote the manuscript. All authors read and approved the final manuscript.

## Competing interests

The authors have declared no competing interests.

## Supplementary material

Supplementary Figure S1 **Cladogram of the LD proteins from algae, higher plants, and mammals** Cladogram was constructed by MEGA6.0 using the neighbor-joining method. GenBank accession numbers of the LD proteins from different organisms are indicated in the parenthesis. Al, *Arabidopsis lyrate*; Ap, *Auxenochlorella protothecoides*; At, *Arabidopsis thaliana*; Bn, *Brassica napus*; Cr, *Chlamydomonas reinhardtii*; Cru, *Capsella rubella*; Csa, *Camelina sativa*; Csu, *Coccomyxa subellipsoidea*; Cv, *Chlorella variabilis*; Cz, *Chromochloris zofingiensis*; Db, *Dunaliella bardawil*; Dp, *Dunaliella parva*; Ds, *Dunaliella salina*; Es, *Ectocarpus siliculosus*; Fs, *Fistulifera solaris*; Hp, *Haematococcus pluvialis*; Hs, *Homo sapiens*; Jc, *Jatropha curcas*; Li, *Lobosphaera incisa*; Ma, *Mesocricetus auratus*; Mm, *Mus musculus*; Mn, *Monoraphidium neglectum*; Mp, *Micromonas pusilla*; Ng, *Nannochloropsis gaditana*; No, *Nannochloropsis oceanica*; Nt, *Nicotiana tabacum*; Pt, *Phaeodactylum tricornutum*; Rn, *Rattus norvegicus*; Rs, *Rhinolophus sinicus*; To, *Thalassiosira oceanica*; Tp, *Thalassiosira pseudonana*; Vc, *Volvox carteri*.

Supplementary Figure S2 **Kyte-Doolittle hydropathy plots using ProtScale** () X axis designates the Kyte-Doolittle hydropathy score and Y axis designates the amino acid position. G indicates the grand average of hydropathy (GRAVY) values of proteins.

Supplementary Figure S3 **Prediction of transmembrane domains by TMHMM Server v. 2.0** () X axis designates the probability of transmembrane domain and Y axis designates the amino acid position.

Supplementary Figure S4 **Expression of LD protein-coding genes under ND conditions** The expression level of the LD protein-coding genes, determined by qPCR, was normalized to that of the endogenous β-actin using the qPCR primers shown in Table S1. Data are expressed as mean ± SD (*n* = 3). An asterisk indicate signiﬁcant difference at a specified time point compared to 0 h (*P* < 0.05; *t*-test). MLDP, major lipid droplet protein; FUP, functionally unknown protein; GULO, l-gulonolactone oxidase.

Supplementary Figure S5 **Alignment of MLDP protein sequences from green algae** MLDP protein sequences were aligned using ClustalX2.1. See Figure S1 for the GenBank accession numbers of the algal MLDP proteins.

Supplementary Figure S6 **Characterization of the *mldp* mutant of *C. reinhardtii* A A.** schematic illustration showing the insertion of paromomycin-resistant cassette into *MLDP* gene of the *mldp* mutant. Green thick arrows indicate exons. The black triangle represents the insertion in the 1st intron. Black and blue arrowheads designate the primers for PCR confirmation of the insertion (F and R) and qPCR of the gene expression (qF and qR), respectively, which are not drawn to scale. **B.** PCR characterization of *Crmldp* and its parent strain, CC-5325. The failure in PCR amplification of *MLDP* indicates the presence of the insertion in *Crmldp*. **C.***MLDP* expression level in *Crmldp* and CC-5325 as determined by qPCR. An asterisk indicates signiﬁcant difference (*P* < 0.05; *t*-test).

Supplementary Figure S7 **Cladogram of the GULO and GLDH proteins from algae, higher plants, and mammals** Cladogram was constructed by MEGA6.0 using the neighbor-joining method. GenBank accession numbers of the GULO and GLDH proteins from different organisms are indicated in the parenthesis.

Supplementary Figure S8 **Cladogram of the lipases from algae, higher plants, and yeast** Cladogram was constructed by MEGA6.0 using the neighbor-joining method. GenBank accession numbers of the lipase proteins from different organisms are indicated in the parenthesis.

Supplementary Table S1 **PCR primers used for the cloning and qPCR of LD protein-coding genes**

Supplementary Dataset S1 **List of all LD proteins of *C. zofingiensis***

Supplementary Dataset S2 **List of *C. zofingiensis* LD proteins detected in both replicates with ≥2 unique peptides**
