# Supplementary figures and images for: Proteomics Analysis of Lipid Droplets from the Oleaginous Alga Chromochloris zofingiensis Reveals Novel Proteins for Lipid Metabolism
Source: Genomics Proteomics Bioinformatics. 2019 Sep 5;17(3):260–72. doi: 10.1016/j.gpb.2019.01.003 (PMC6818385; doi:10.1016/j.gpb.2019.01.003)

## Slide 1
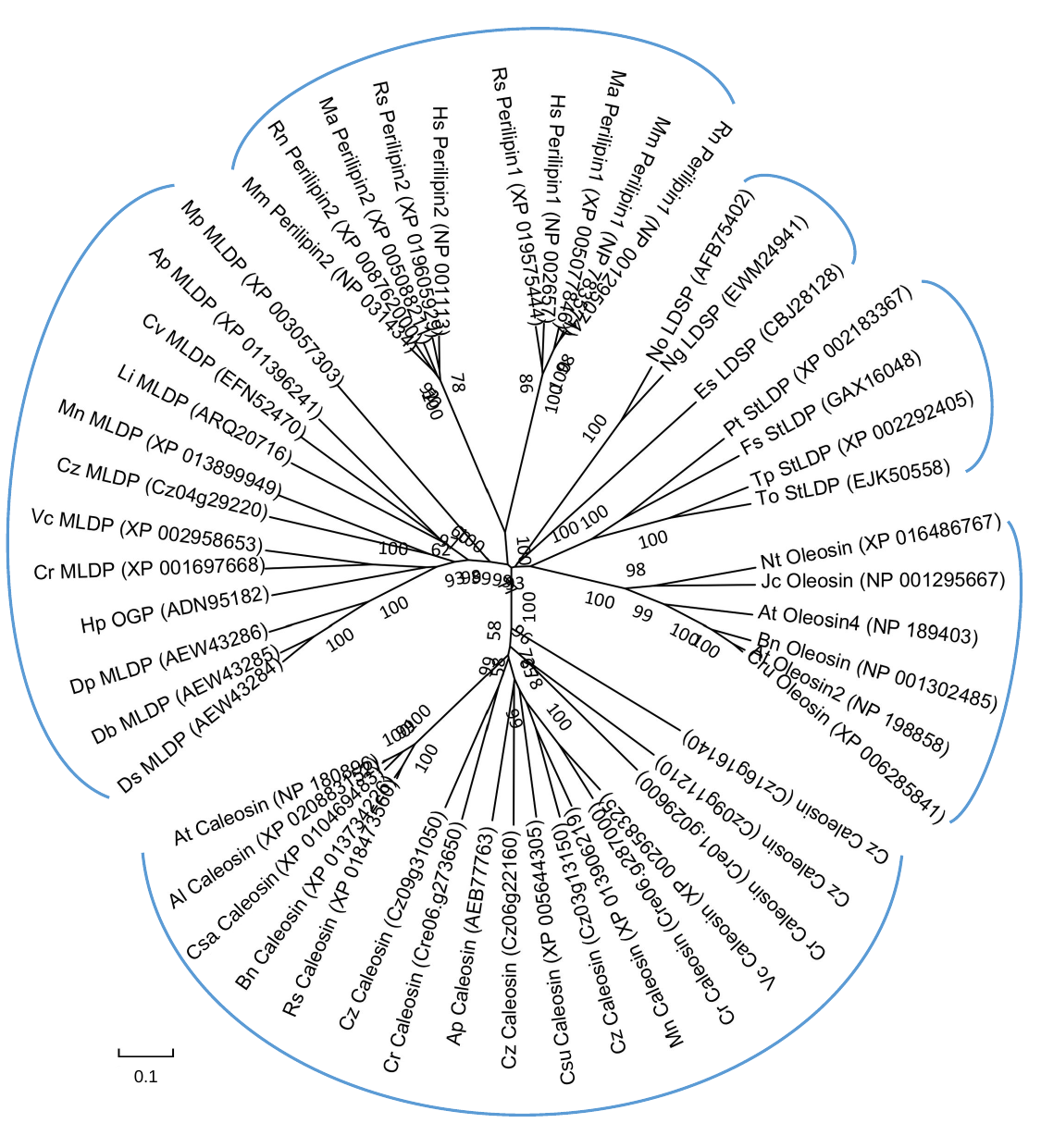

Csa Caleosin
Csa
Csu
0.1

Supplement: Supplementary Figure S1 — Cladogram of the LD proteins from algae, higher plants, and mammals Cladogram was constructed by MEGA6.0 using the neighbor-joining method. GenBank accession numbers of the LD proteins from different organisms are indicated in the parenthesis. Al, Arabidopsis lyrate; Ap, Auxenochlorella protothecoides; At, Arabidopsis thaliana; Bn, Brassica napus; Cr, Chlamydomonas reinhardtii; Cru, Capsella rubella; Csa, Camelina sativa; Csu, Coccomyxa subellipsoidea; Cv, Chlorella variabilis; Cz, Chromochloris zofingiensis; Db, Dunaliella bardawil; Dp, Dunaliella parva; Ds, Dunaliella salina; Es, Ectocarpus siliculosus; Fs, Fistulifera solaris; Hp, Haematococcus pluvialis; Hs, Homo sapiens; Jc, Jatropha curcas; Li, Lobosphaera incisa; Ma, Mesocricetus auratus; Mm, Mus musculus; Mn, Monoraphidium neglectum; Mp, Micromonas pusilla; Ng, Nannochloropsis gaditana; No, Nannochloropsis oceanica; Nt, Nicotiana tabacum; Pt, Phaeodactylum tricornutum; Rn, Rattus norvegicus; Rs, Rhinolophus sinicus; To, Thalassiosira oceanica; Tp, Thalassiosira pseudonana; Vc, Volvox carteri. [file mmc1.pptx]
