# Supplementary material for: Proteomics Analysis of Lipid Droplets from the Oleaginous Alga Chromochloris zofingiensis Reveals Novel Proteins for Lipid Metabolism
Source: Genomics Proteomics Bioinformatics. 2019 Sep 5;17(3):260–72. doi: 10.1016/j.gpb.2019.01.003 (PMC6818385; doi:10.1016/j.gpb.2019.01.003)
Supplement: Supplementary Figure S2 — Kyte-Doolittle hydropathy plots using ProtScale () X axis designates the Kyte-Doolittle hydropathy score and Y axis designates the amino acid position. G indicates the grand average of hydropathy (GRAVY) values of proteins. [file mmc2.pptx]

## Slide 1
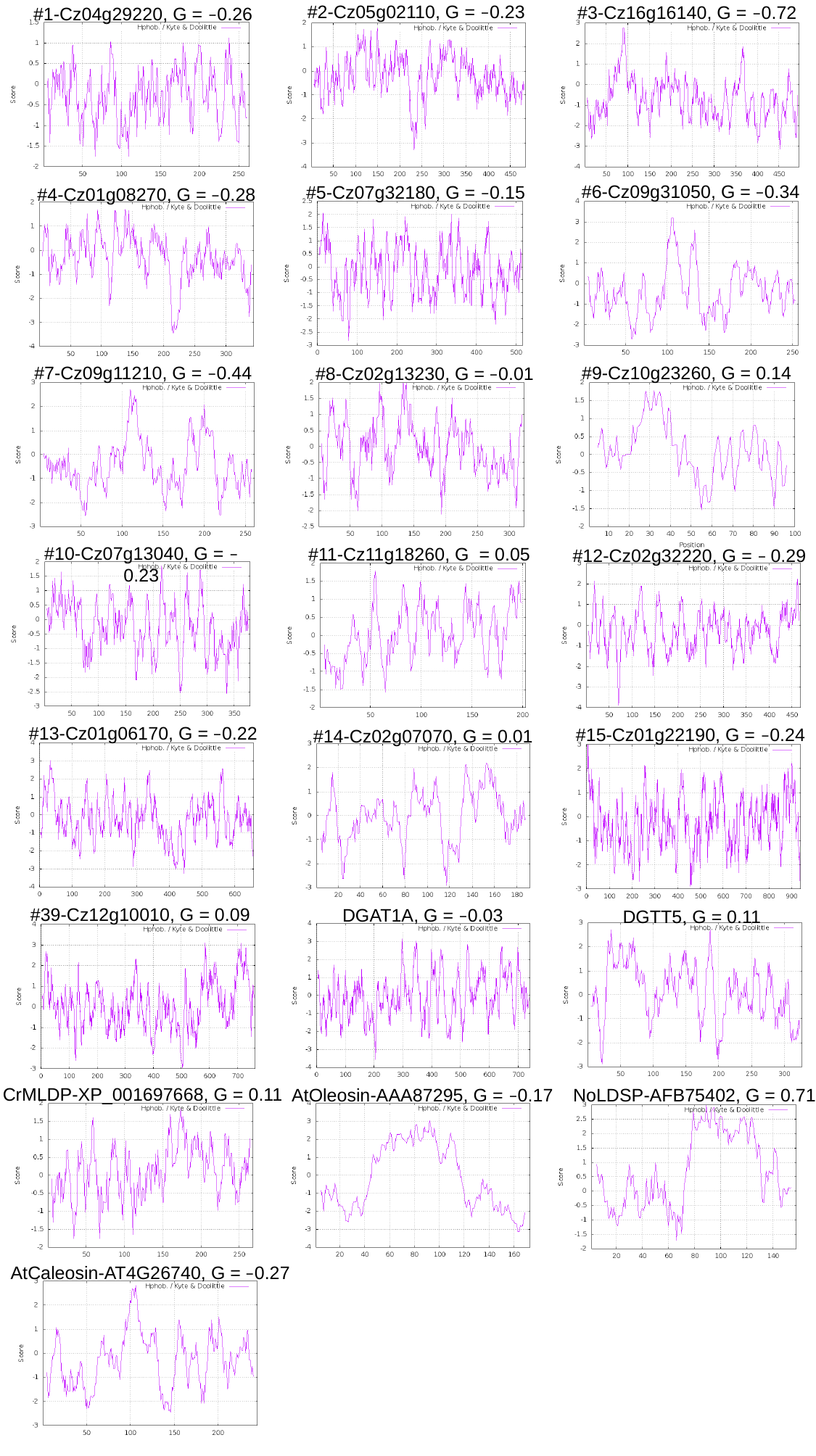

#2-Cz05g02110, G = –0.23
#3-Cz16g16140, G = –0.72
#1-Cz04g29220, G = –0.26
#6-Cz09g31050, G = –0.34
#5-Cz07g32180, G = –0.15
#4-Cz01g08270, G = –0.28
#7-Cz09g11210, G = –0.44
#9-Cz10g23260, G = 0.14
#8-Cz02g13230, G = –0.01
#10-Cz07g13040, G = – 0.23
#11-Cz11g18260, G = 0.05
#12-Cz02g32220, G = – 0.29
#13-Cz01g06170, G = –0.22
#15-Cz01g22190, G = –0.24
#14-Cz02g07070, G = 0.01
#39-Cz12g10010, G = 0.09
DGAT1A, G = –0.03
DGTT5, G = 0.11
CrMLDP-XP_001697668, G = 0.11
NoLDSP-AFB75402, G = 0.71
AtOleosin-AAA87295, G = –0.17
AtCaleosin-AT4G26740, G = –0.27
