# Supplementary material for: Proteomics Analysis of Lipid Droplets from the Oleaginous Alga Chromochloris zofingiensis Reveals Novel Proteins for Lipid Metabolism
Source: Genomics Proteomics Bioinformatics. 2019 Sep 5;17(3):260–72. doi: 10.1016/j.gpb.2019.01.003 (PMC6818385; doi:10.1016/j.gpb.2019.01.003)
Supplement: Supplementary Figure S3 — Prediction of transmembrane domains by TMHMM Server v. 2.0 () X axis designates the probability of transmembrane domain and Y axis designates the amino acid position. [file mmc3.pptx]

## Slide 1
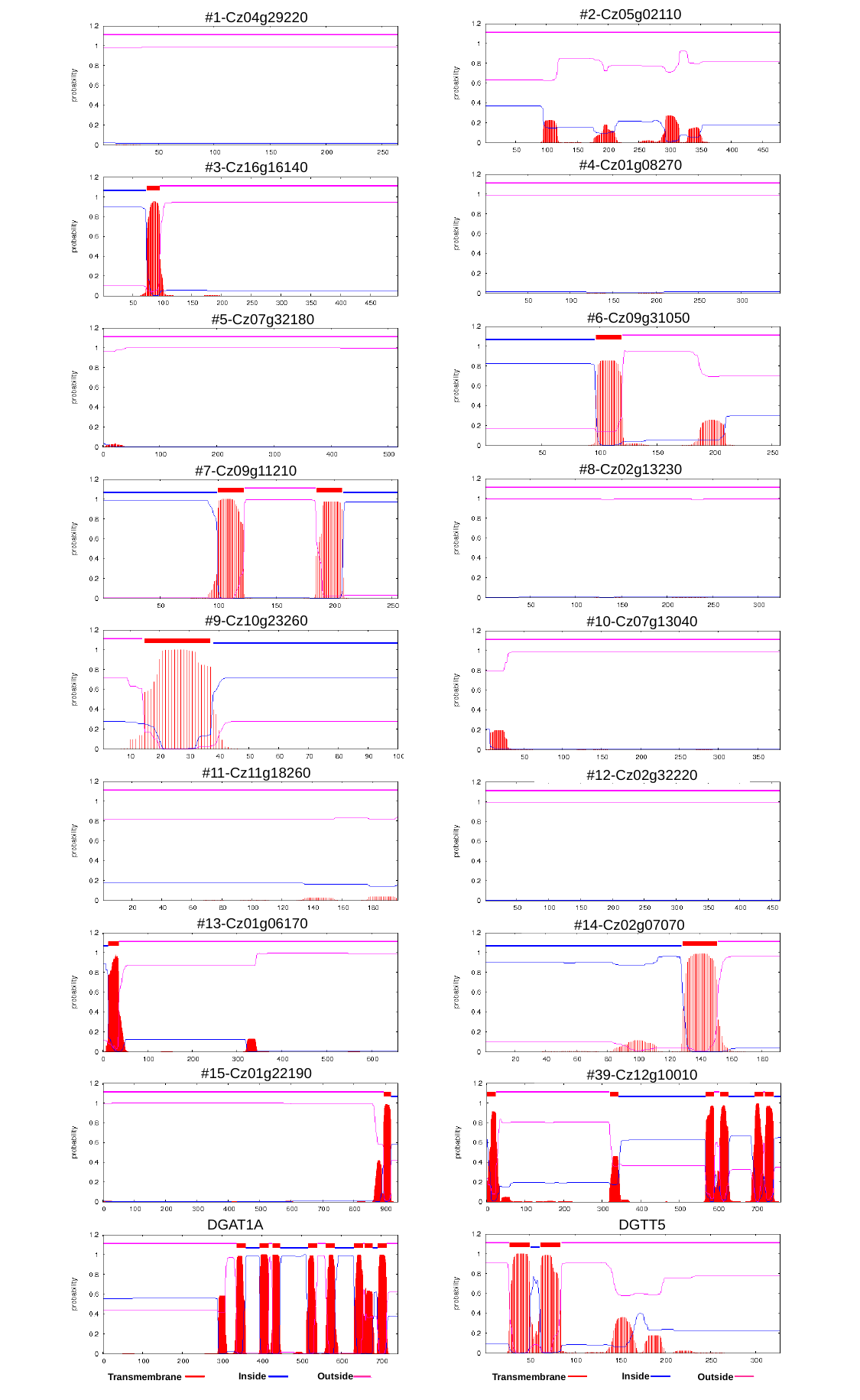

#2-Cz05g02110
#1-Cz04g29220
#4-Cz01g08270
#3-Cz16g16140
#6-Cz09g31050
#5-Cz07g32180
#8-Cz02g13230
#7-Cz09g11210
#9-Cz10g23260
#10-Cz07g13040
#11-Cz11g18260
#12-Cz02g32220
#13-Cz01g06170
#14-Cz02g07070
#15-Cz01g22190
#39-Cz12g10010
DGAT1A
DGTT5
Inside
Outside
Inside
Transmembrane
Transmembrane
Outside
