# Supplementary material for: Proteomics Analysis of Lipid Droplets from the Oleaginous Alga Chromochloris zofingiensis Reveals Novel Proteins for Lipid Metabolism
Source: Genomics Proteomics Bioinformatics. 2019 Sep 5;17(3):260–72. doi: 10.1016/j.gpb.2019.01.003 (PMC6818385; doi:10.1016/j.gpb.2019.01.003)
Supplement: Supplementary Figure S4 — Expression of LD protein-coding genes under ND conditions The expression level of the LD protein-coding genes, determined by qPCR, was normalized to that of the endogenous β-actin using the qPCR primers shown in Table S1. Data are expressed as mean ± SD (n = 3). An asterisk indicate signiﬁcant difference at a specified time point compared to 0 h (P < 0.05; t-test). MLDP, major lipid droplet protein; FUP, functionally unknown protein; GULO, l-gulonolactone oxidase. [file mmc4.pptx]

## Slide 1
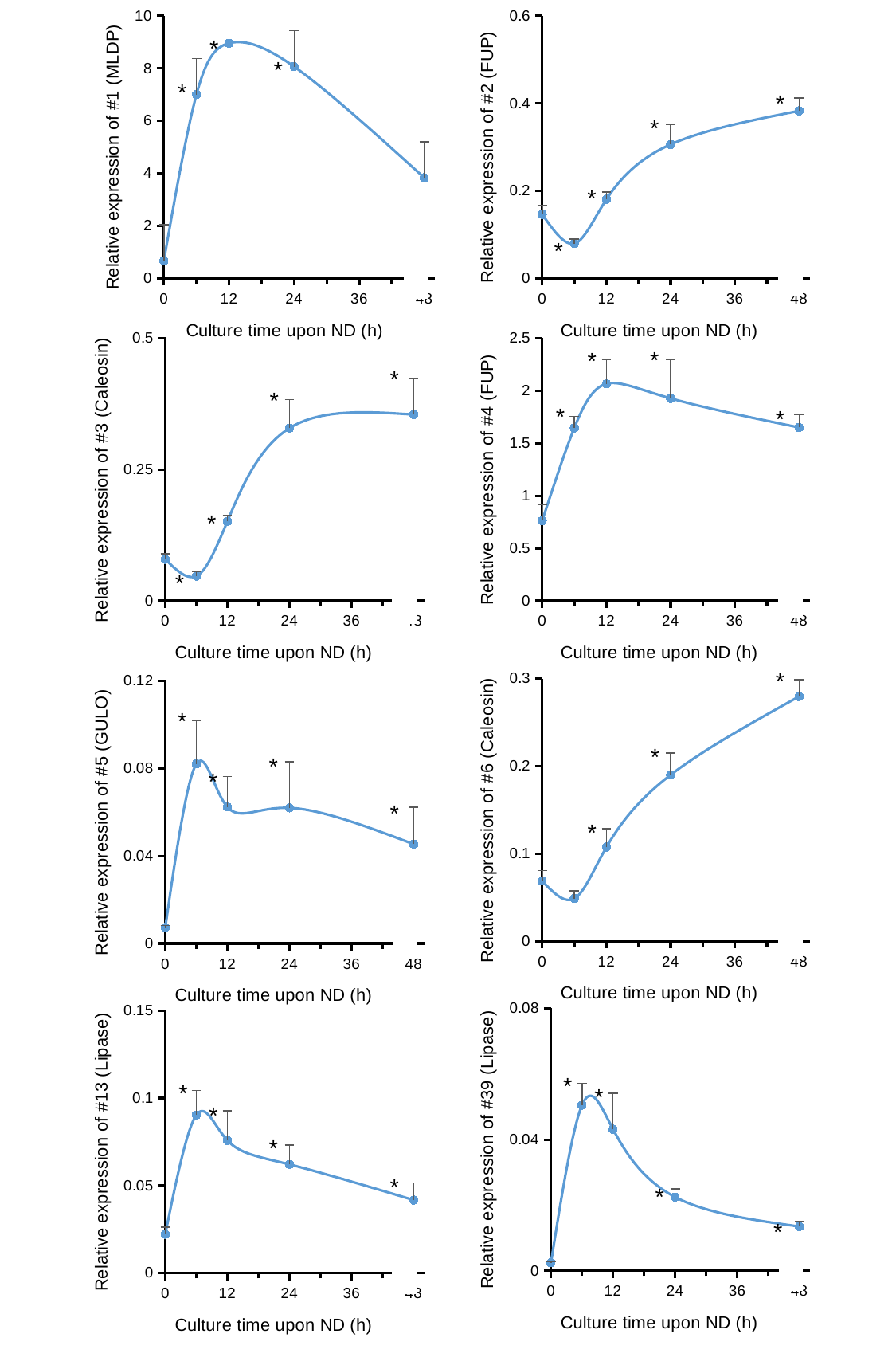

### Chart
| Category | |
|---|---|
### Chart
| Category | |
|---|---|*
*
*
*
*
*
*
### Chart
| Category | |
|---|---|
### Chart
| Category | |
|---|---|*
*
*
*
*
*
*
*
*
### Chart
| Category | |
|---|---|
### Chart
| Category | |
|---|---|*
*
*
*
*
*
### Chart
| Category | |
|---|---|
### Chart
| Category | |
|---|---|*
*
*
*
*
*
*
*
