# Supplementary material for: Proteomics Analysis of Lipid Droplets from the Oleaginous Alga Chromochloris zofingiensis Reveals Novel Proteins for Lipid Metabolism
Source: Genomics Proteomics Bioinformatics. 2019 Sep 5;17(3):260–72. doi: 10.1016/j.gpb.2019.01.003 (PMC6818385; doi:10.1016/j.gpb.2019.01.003)
Supplement: Supplementary Figure S5 — Alignment of MLDP protein sequences from green algae MLDP protein sequences were aligned using ClustalX2.1. See Figure S1 for the GenBank accession numbers of the algal MLDP proteins. [file mmc5.pptx]

## Slide 1
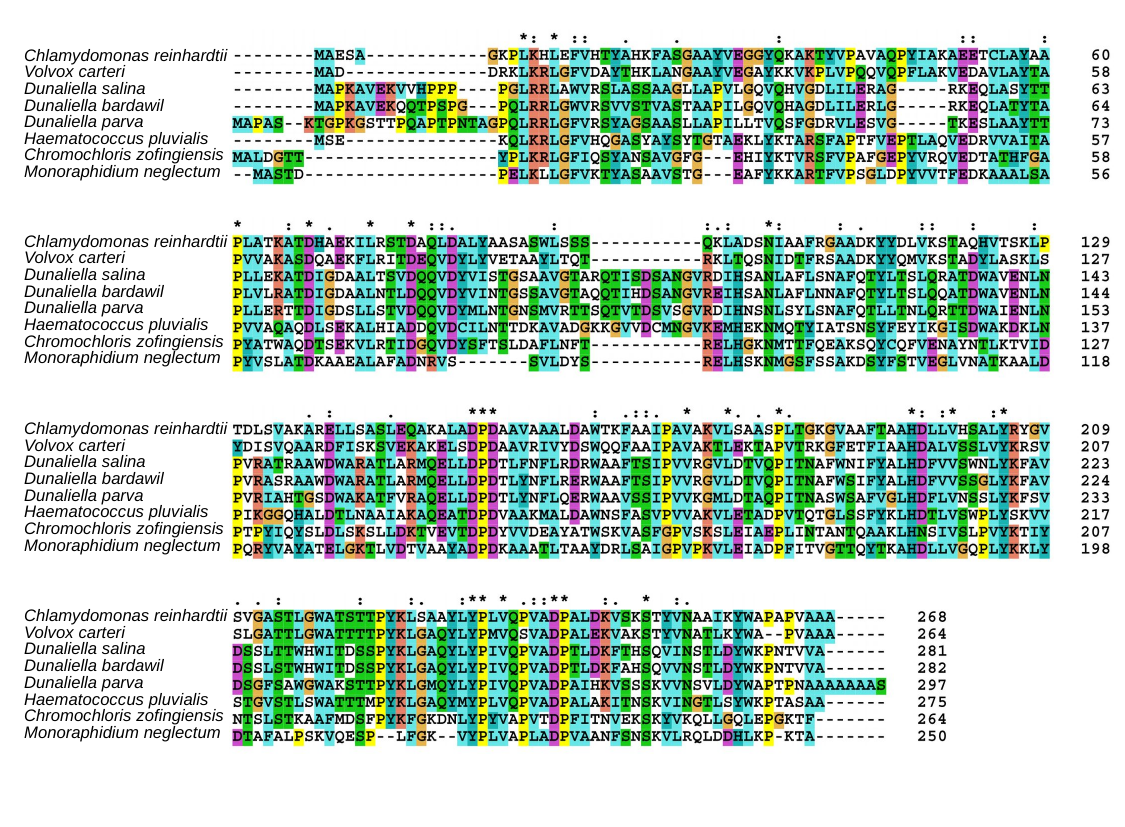

Chlamydomonas reinhardtii
Volvox carteri
Dunaliella salina
Dunaliella bardawil
Dunaliella parva
Haematococcus pluvialis
Chromochloris zofingiensis
Monoraphidium neglectum
Chlamydomonas reinhardtii
Volvox carteri
Dunaliella salina
Dunaliella bardawil
Dunaliella parva
Haematococcus pluvialis
Chromochloris zofingiensis
Monoraphidium neglectum
Chlamydomonas reinhardtii
Volvox carteri
Dunaliella salina
Dunaliella bardawil
Dunaliella parva
Haematococcus pluvialis
Chromochloris zofingiensis
Monoraphidium neglectum
Chlamydomonas reinhardtii
Volvox carteri
Dunaliella salina
Dunaliella bardawil
Dunaliella parva
Haematococcus pluvialis
Chromochloris zofingiensis
Monoraphidium neglectum
