# Supplementary material for: Proteomics Analysis of Lipid Droplets from the Oleaginous Alga Chromochloris zofingiensis Reveals Novel Proteins for Lipid Metabolism
Source: Genomics Proteomics Bioinformatics. 2019 Sep 5;17(3):260–72. doi: 10.1016/j.gpb.2019.01.003 (PMC6818385; doi:10.1016/j.gpb.2019.01.003)
Supplement: Supplementary Figure S6 — Characterization of the mldp mutant of C. reinhardtii A A. schematic illustration showing the insertion of paromomycin-resistant cassette into MLDP gene of the mldp mutant. Green thick arrows indicate exons. The black triangle represents the insertion in the 1st intron. Black and blue arrowheads designate the primers for PCR confirmation of the insertion (F and R) and qPCR of the gene expression (qF and qR), respectively, which are not drawn to scale. B. PCR characterization of Crmldp and its parent strain, CC-5325. The failure in PCR amplification of MLDP indicates the presence of the insertion in Crmldp. C.MLDP expression level in Crmldp and CC-5325 as determined by qPCR. An asterisk indicates signiﬁcant difference (P < 0.05; t-test). [file mmc6.pptx]

## Slide 1
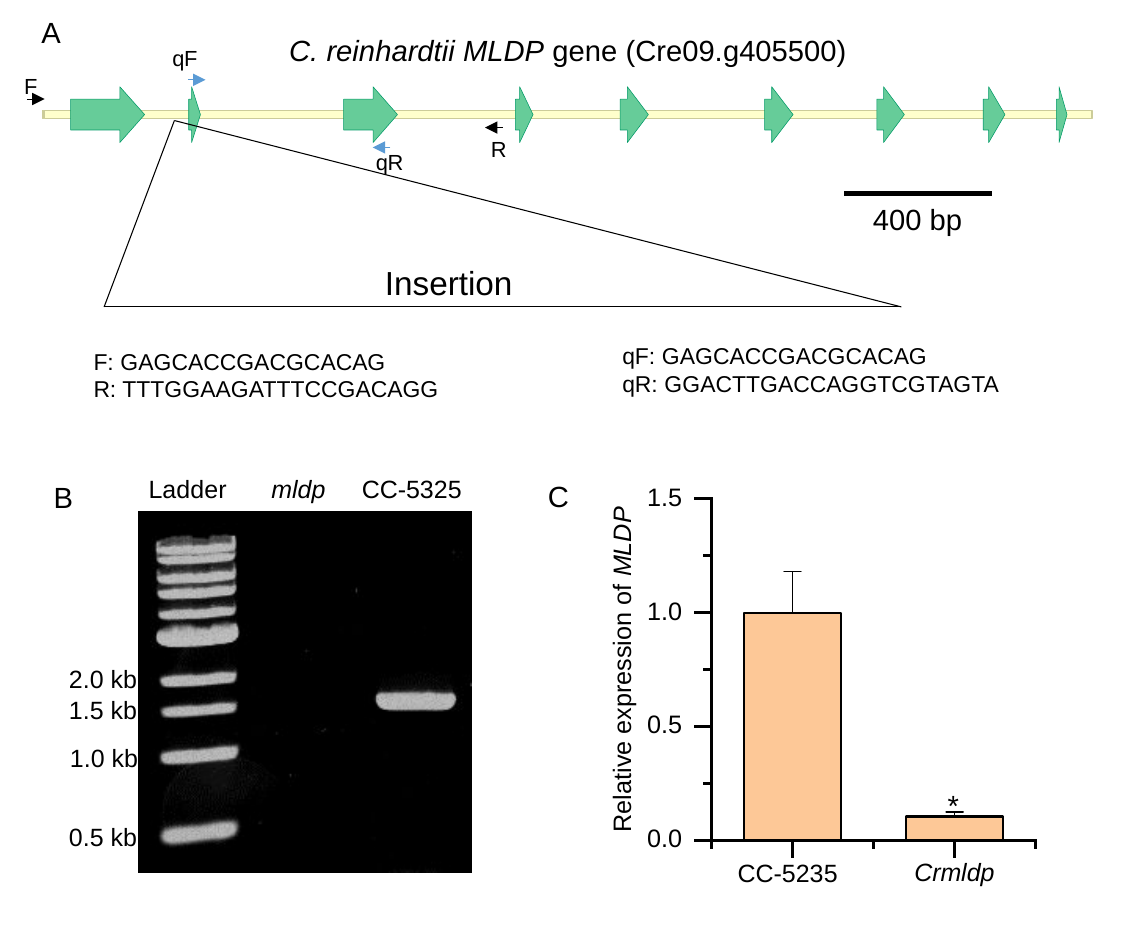

A
C. reinhardtii MLDP gene (Cre09.g405500)
qF
F
R
qR
400 bp
Insertion
qF: GAGCACCGACGCACAG
qR: GGACTTGACCAGGTCGTAGTA
F: GAGCACCGACGCACAG
R: TTTGGAAGATTTCCGACAGG
Ladder
mldp
CC-5325
C
B
2.0 kb
1.5 kb
1.0 kb
*
0.5 kb
Crmldp
CC-5235

## Slide 2
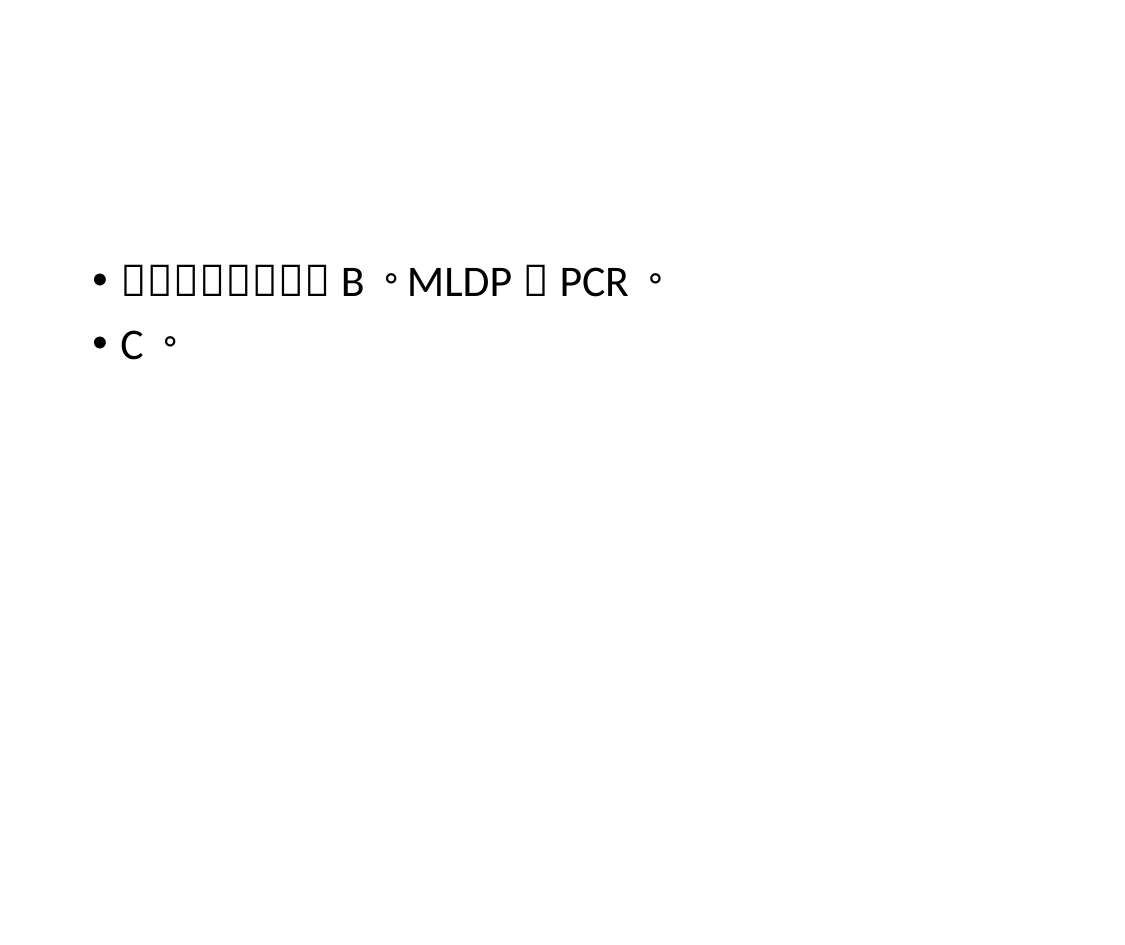

#
谢谢老师，我们把B图下标移动到上标。最右道是MLDP的PCR出的条带。
C图是基因。
