# Supplementary material for: Proteomics Analysis of Lipid Droplets from the Oleaginous Alga Chromochloris zofingiensis Reveals Novel Proteins for Lipid Metabolism
Source: Genomics Proteomics Bioinformatics. 2019 Sep 5;17(3):260–72. doi: 10.1016/j.gpb.2019.01.003 (PMC6818385; doi:10.1016/j.gpb.2019.01.003)
Supplement: Supplementary Figure S7 — Cladogram of the GULO and GLDH proteins from algae, higher plants, and mammals Cladogram was constructed by MEGA6.0 using the neighbor-joining method. GenBank accession numbers of the GULO and GLDH proteins from different organisms are indicated in the parenthesis. [file mmc7.pptx]

## Slide 1
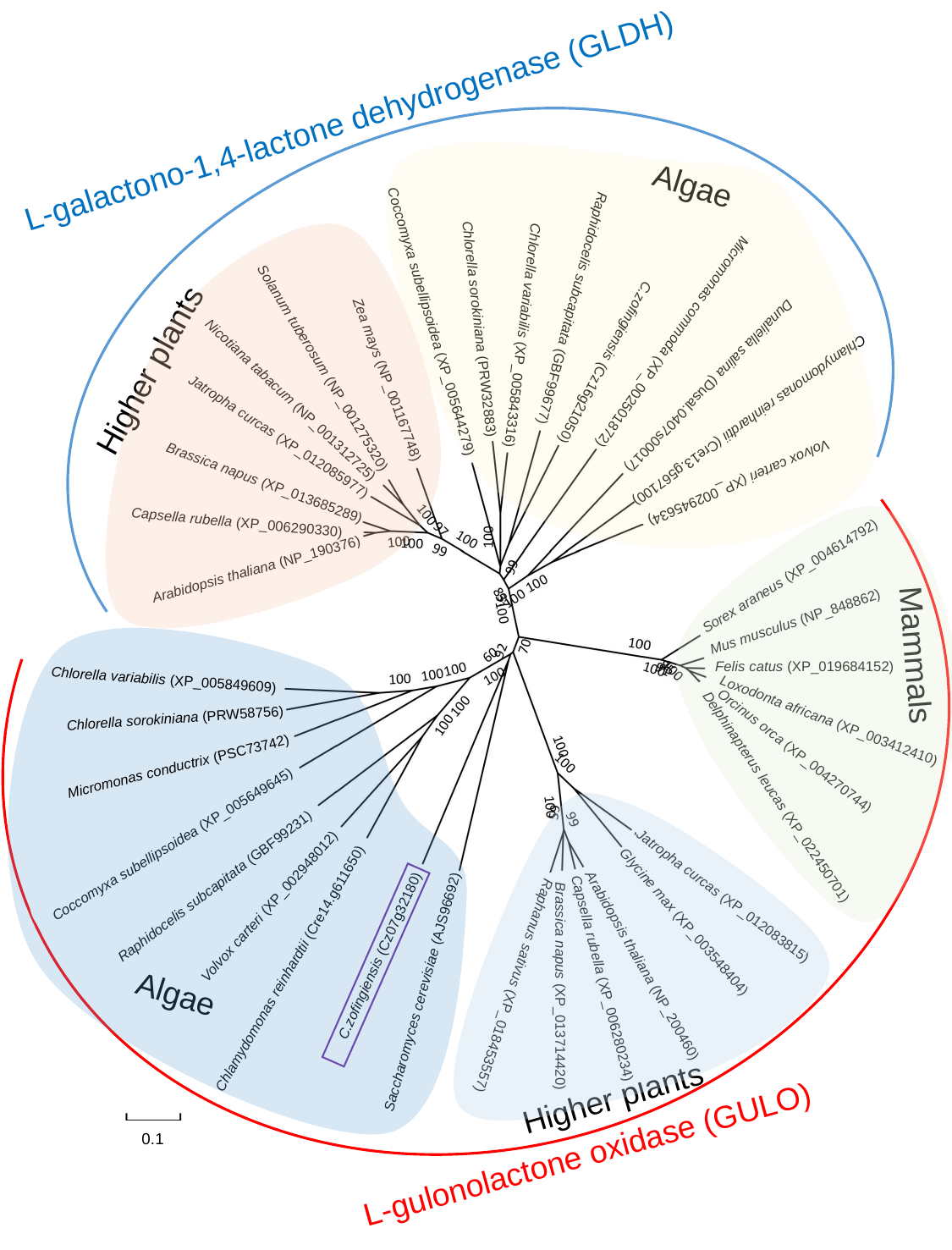

L-galactono-1,4-lactone dehydrogenase (GLDH)
Algae
Raphidocelis subcapitata (GBF99677)
Coccomyxa subellipsoidea (XP_005644279)
Chlorella sorokiniana (PRW32883)
Chlorella variabilis (XP_005843316)
Micromonas commoda (XP_002501872)
Higher plants
C.zofingiensis (Cz16g21050)
Solanum tuberosum (NP_001275320)
Zea mays (NP_001167748)
Dunaliella salina (Dusal.0407s00017)
Nicotiana tabacum (NP_001312725)
Chlamydomonas reinhardtii (Cre13.g567100)
Jatropha curcas (XP_012085977)
Brassica napus (XP_013685289)
Volvox carteri (XP_002945634)
Capsella rubella (XP_006290330)
Arabidopsis thaliana (NP_190376)
Sorex araneus (XP_004614792)
Mus musculus (NP_848862)
Mammals
Felis catus (XP_019684152)
Chlorella variabilis (XP_005849609)
Chlorella sorokiniana (PRW58756)
Loxodonta africana (XP_003412410)
Orcinus orca (XP_004270744)
Micromonas conductrix (PSC73742)
Delphinapterus leucas (XP_022450701)
Coccomyxa subellipsoidea (XP_005649645)
Raphidocelis subcapitata (GBF99231)
Jatropha curcas (XP_012083815)
Volvox carteri (XP_002948012)
Glycine max (XP_003548404)
C.zofingiensis (Cz07g32180)
Arabidopsis thaliana (NP_200460)
Chlamydomonas reinhardtii (Cre14.g611650)
Capsella rubella (XP_006280234)
Algae
Raphanus sativus (XP_018453557)
Brassica napus (XP_013714420)
Saccharomyces cerevisiae (AJS96692)
Higher plants
0.1
L-gulonolactone oxidase (GULO)
