# Supplementary material for: Proteomics Analysis of Lipid Droplets from the Oleaginous Alga Chromochloris zofingiensis Reveals Novel Proteins for Lipid Metabolism
Source: Genomics Proteomics Bioinformatics. 2019 Sep 5;17(3):260–72. doi: 10.1016/j.gpb.2019.01.003 (PMC6818385; doi:10.1016/j.gpb.2019.01.003)
Supplement: Supplementary Figure S8 — Cladogram of the lipases from algae, higher plants, and yeast Cladogram was constructed by MEGA6.0 using the neighbor-joining method. GenBank accession numbers of the lipase proteins from different organisms are indicated in the parenthesis. [file mmc8.pptx]

## Slide 1
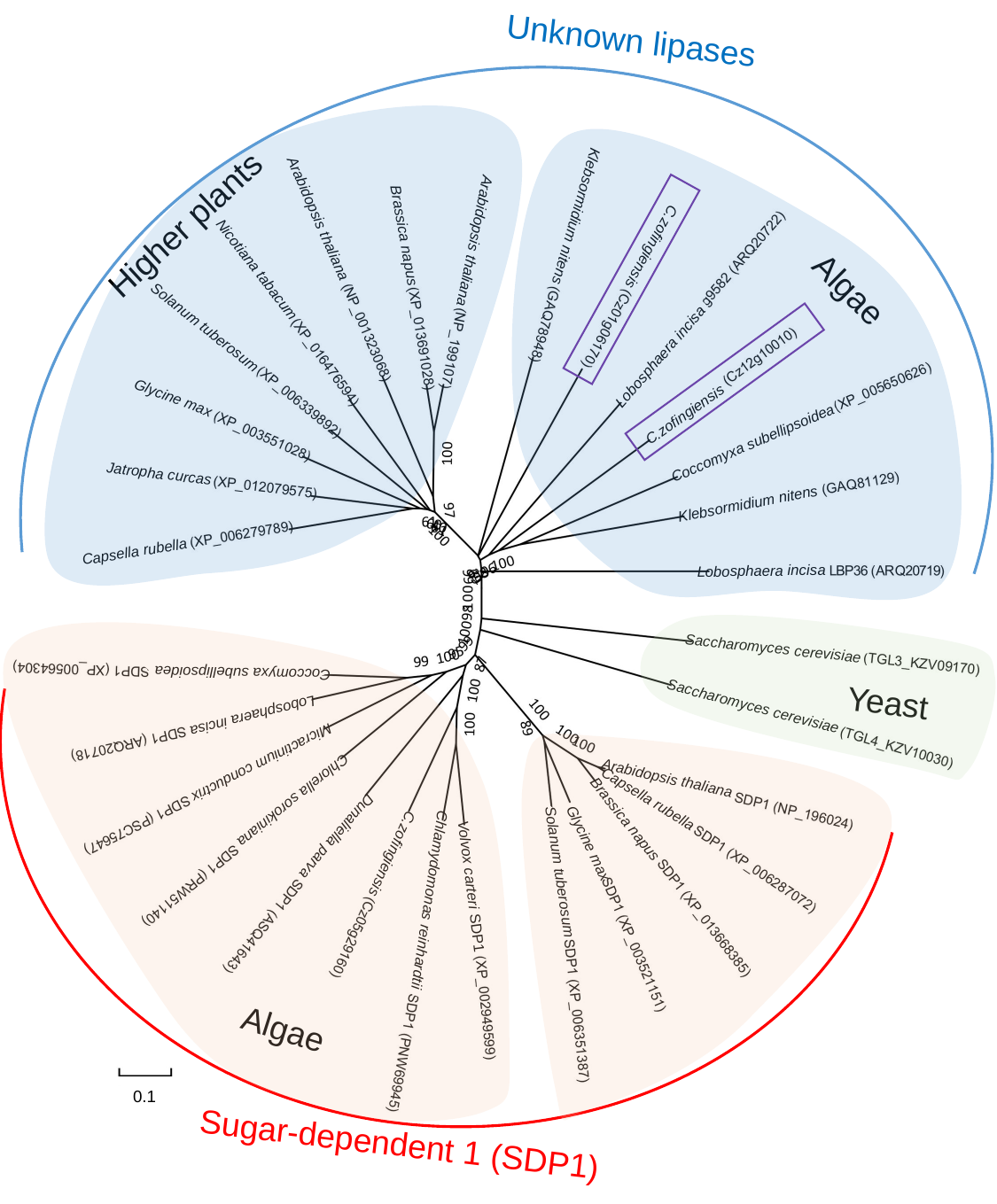

Unknown lipases
Higher plants
Klebsormidium nitens
Arabidopsis thaliana
Brassica napus
Arabidopsis thaliana
C.zofingiensis
Nicotiana tabacum
Algae
Solanum tuberosum
Lobosphaera incisa
Glycine max
C.zofingiensis
Coccomyxa subellipsoidea
Jatropha curcas
Klebsormidium nitens
Capsella rubella
Lobosphaera incisa
Saccharomyces cerevisiae
Coccomyxa subellipsoidea
Yeast
Saccharomyces cerevisiae
Lobosphaera incisa
Micractinium conductrix
Arabidopsis thaliana
Capsella rubella
Chlorella sorokiniana
Brassica napus
Dunaliella parva
Glycine max
C.zofingiensis
Solanum tuberosum
Volvox carteri SDP1
Chlamydomonas reinhardtii
Algae
0.1
Sugar-dependent 1 (SDP1)
