# Supplementary material for: Proteomics Analysis of Lipid Droplets from the Oleaginous Alga Chromochloris zofingiensis Reveals Novel Proteins for Lipid Metabolism
Source: Genomics Proteomics Bioinformatics. 2019 Sep 5;17(3):260–72. doi: 10.1016/j.gpb.2019.01.003 (PMC6818385; doi:10.1016/j.gpb.2019.01.003)
Supplement: Supplementary Table S1 — PCR primers used for the cloning and qPCR of LD protein-coding genes [file mmc9.docx]

**Table S1 PCR primers used for the cloning and qPCR of LD protein-coding genes**

| **Gene** | **Forward (5'-3')** | **Reverse (5'-3')** |
| --- | --- | --- |
| *For gene cloning* | | |
| #1 (MLDP) | CCGGAATTCATGGCTCTTGACGGCACCACC | CCGCTCGAGGAAGGTCTTGCCTGGCTCCAG |
| #2 (FUP) | CCGGAATTCATGACAGATCTCAGGGTTAAC | CCGCTCGAGGTAAGCATTGGTGGTGGTAGT |
| #3 (Caleosin) | CCGGAATTCATGTCGTACATGACCAACCCC | CCGCTCGAGCAACACCCCTTCTCTCTTTGC |
| #4 (FUP) | CCGGAATTCATGGCAACGGAAACTCCAATT | CCGCTCGAGAGCCAGCACTTTCTCACCACG |
| #5 (GULO) | CCGGAATTCATGGCCATGGGTGCTTCTCCA | CCGCTCGAGTAGCATTTTGTCACCACCGCC |
| #6 (Caleosin) | *GCCGCCAGTGTGCTGGAATTC*ATGTTGCTGCCTATTCTAAGAAACC | *GCCCTTGCTCACCATCTCGAG*GCCCCCCGCAGCAGCAGC |
| #8 (RDH) | *GCCGCCAGTGTGCTGGAATTC*ATGGGTCTCCTGGGCTGGT | *GCCCTTGCTCACCATCTCGAG*GGCTAGCTGTCCGACAGCC |
| #13 (Lipase) | *GCCGCCAGTGTGCTGGAATTC*ATGACGACTTGGGACGATCTGT | *GCCCTTGCTCACCATCTCGAG*GCGTGAGTGCTCGTGTGGG |
| #39 (Lipase) | *GCCGCCAGTGTGCTGGAATTC*ATGTGGATTGCAGCGTGGC | *GCCCTTGCTCACCATCTCGAG*CCAGTCCTGTGGAGCGCTG |
| *For qPCR* | | |
| #1 (MLDP) | GCTCCTTCGTTCCTGCATTT | CGCAGCACCTTCTCGCTAGT |
| #2 (FUP) | CAGCCTGGAGAATGGCAAGA | ACCCATCCACATGCCATTG |
| #3 (Caleosin) | TGGCCACTGGACACATTCTG | GCCGCTGTACCAGCTGAAG |
| #4 (FUP) | CCCTTGGAGTCTGGTCAGATTG | CACATACCCCATGCCAAAGC |
| #5 (GULO) | TCAACTTGCACCCGCACTAC | GCACTGTCACCAGAGCAGTTGT |
| #6 (Caleosin) | CTGGCATGGTGACCAAGGA | GCAGCACCCCCTTCTTTCA |
| #8 (RDH) | AGACCCCTGGTGGAGTTGTG | ACCATACCCTGGCAAAGAACA |
| #13 (Lipase) | GCACTGCATGGCTGACAGATT | TGCTTGAAGCCTGTGTGGAA |
| β-actin | GCTGGCATTCACGACACAAC | TGCCACCACCTTGATCTTCA |

*Note*：Underlined sequences indicate the restriction enzyme sites, while the italic underlined sequences designate the homologous sequences to the vector for In-Fusion cloning. See Dataset S1 for more information about the LD protein-coding genes shown in the first column. MLDP, major lipid droplet protein; FUP, function unknown protein; GULO, L-gulonolactone oxidase; RDH, retinol dehydrogenase
